# Disentangling cobionts and contamination in long-read genomic data using sequence composition

**DOI:** 10.1093/g3journal/jkae187

**Published:** 2024-08-16

**Authors:** Claudia C Weber

**Affiliations:** Tree of Life, Wellcome Sanger Institute, Wellcome Genome Campus, Hinxton CB10 1SA, UK

**Keywords:** variational autoencoder, data visualization, unsupervised learning, long-read sequencing

## Abstract

The recent acceleration in genome sequencing targeting previously unexplored parts of the tree of life presents computational challenges. Samples collected from the wild often contain sequences from several organisms, including the target, its cobionts, and contaminants. Effective methods are therefore needed to separate sequences. Though advances in sequencing technology make this task easier, it remains difficult to taxonomically assign sequences from eukaryotic taxa that are not well represented in databases. Therefore, reference-based methods alone are insufficient. Here, I examine how we can take advantage of differences in sequence composition between organisms to identify symbionts, parasites, and contaminants in samples, with minimal reliance on reference data. To this end, I explore data from the Darwin Tree of Life project, including hundreds of high-quality HiFi read sets from insects. Visualizing two-dimensional representations of read tetranucleotide composition learned by a variational autoencoder can reveal distinct components of a sample. Annotating the embeddings with additional information, such as coding density, estimated coverage, or taxonomic labels allows rapid assessment of the contents of a dataset. The approach scales to millions of sequences, making it possible to explore unassembled read sets, even for large genomes. Combined with interactive visualization tools, it allows a large fraction of cobionts reported by reference-based screening to be identified. Crucially, it also facilitates retrieving genomes for which suitable reference data are absent.

## Teaser

Samples collected for genomic sequencing often contain genetic material from several organisms. Determining if a given sequence represents contamination can be difficult without suitable reference data. However, sequence composition can differ vastly between genomes. This work explores how 2D representations of composition can help separate genomic long reads from different sources. A Variational Autoencoder (a generative model based on a neural network) provides an effective framework for embedding millions of reads and identifying sequences from parasites, symbionts, and contaminants.

## Introduction

Recent advances in sequencing technology are driving large-scale reference genome production for a wide range of organisms, especially taxa that have not been sequenced extensively. A key aim of these efforts is to better understand the evolution of these species, along with their roles in ecosystems (Lewin *et al.* 2018; McKenna *et al.* 2021; Blaxter *et al.* 2022; Darwin Tree of Life Project Consortium 2022). In addition to the target genome, wild-sourced samples from a species of interest often contain additional genetic material from organelles, cobionts (such as the microbiome, symbionts, and parasites), and environmental contaminants. The presence of nontarget sequence can be an obstacle to generating a reliable assembly for the target organism, as evidenced by many published genomes that contain contamination (Merchant *et al.* 2014; Koutsovoulos *et al.* 2016; Challis *et al.* 2020; Francois *et al.* 2020). Contaminated assemblies can compromise downstream analyses and lead to inaccurate biological conclusions. On the other hand, these data present an opportunity to characterize ecological associations between organisms. Given suitable computational tools, we can also generate reference-quality genomes for cobionts, including unculturable endosymbionts (Kumar and Blaxter 2011; Vancaester and Blaxter 2023).

Efforts such as the Darwin Tree of Life Project (DToL) (Darwin Tree of Life Project Consortium 2022), which aims to sequence 70,000 eukaryotic genomes, provide an unprecedented opportunity to study the evolution of a wide range of organisms and their interacting partners. The high-quality long-read sequencing data being generated as part of the project ought to allow sequences from different sources to be more easily disentangled. In addition to improving assembly contiguity, the reads themselves can be more accurately taxonomically classified (Wenger *et al.* 2019; Portik *et al.* 2022). The latter approach is commonly applied to metagenomic analysis of bacterial communities. However, sequences from eukaryotes including animals, plants, fungi, and protists remain more challenging to work with than their prokaryotic cousins, which have been sequenced more extensively.

Reference-based methods for sequence binning rely on comparisons to databases, which are often contaminated (Breitwieser *et al.* 2019; Bagheri *et al.* 2020; Challis *et al.* 2020; Francois *et al.* 2020; Steinegger and Salzberg 2020; Orakov *et al.* 2021), leading to incorrect assignments. In addition, a sufficiently closely related reference may be unavailable for many organisms—especially when considering parts of the tree of life that have not yet been widely explored. The problem becomes even more acute for sequences with high rates of evolutionary divergence. Accordingly, sequences from genomes with a low density of evolutionarily constrained sites, as found in many multicellular organisms, can be difficult to taxonomically assign. Supervised neural network classifiers have similar limitations, as they are, by definition, trained on sequences that have already been discovered. Performance on “out of distribution” samples that do not resemble any data the model has encountered before can therefore be unreliable (Ponsero and Hurwitz 2019; Ren *et al.* 2019).

How can we reliably separate sequences despite database gaps? Approaches that take advantage of inherent differences in sequence composition between organisms can be helpful. For example, BlobToolKit (BTK) (Challis *et al.* 2020) helps to interactively visualize and extract groups of sequences with differing GC content and coverage. Since GC content provides a limited summary of composition and is not always sufficient to distinguish different organisms (Teeling *et al.* 2004), short substrings (k-mers) can also be used for unsupervised binning. Separating contigs on the basis of k-mer frequencies and coverage, often with the help of dimensionality reduction, is well established in metagenomics (Dick *et al.* 2009; Alneberg *et al.* 2014; Nissen *et al.* 2021). However, the performance of existing tools on mixtures of sequences that include organisms with substantial intragenomic heterogeneity has yet to be explored. Although base composition varies substantially between microbial genomes (Sueoka 1962; Warnecke *et al.* 2009), they are relatively compact and internally homogeneous. Meanwhile, composition in larger genomes is influenced by the spatial distribution of genomic features such as coding sequences, repetitive elements (Chakraborty *et al.* 2021; Hoyt *et al.* 2022), and recombination breakpoints (Galtier *et al.* 2001; Weber *et al.* 2014; Galtier 2021).

Composition-based clustering of unassembled sequencing reads has also received less attention (Wickramarachchi and Lin 2022), despite its potential to inform the sequencing and assembly process. The ability to rapidly assess the contents of a read set would provide an opportunity to gauge the quality of a sample prior to assembly—including whether the target genome is present at sufficient coverage. Given the high accuracy of HiFi reads (Wenger *et al.* 2019), it might be tempting to treat them as short contigs. However, the number of sequences in a read set can run into the millions, creating computational challenges. In addition, differences in sequencing coverage can be helpful in separating different components of a sample (Alneberg *et al.* 2014; Challis *et al.* 2020; Nissen *et al.* 2021; Wickramarachchi and Lin 2022), but this metric usually depends on the availability of an assembly.

**Fig. 1.**
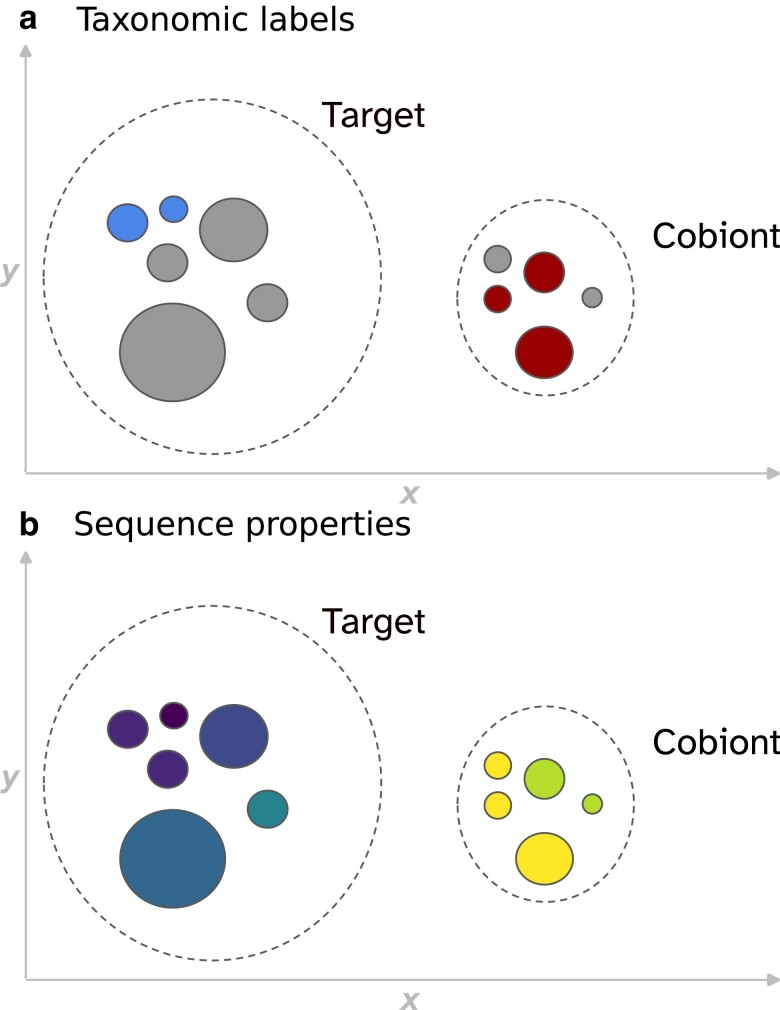
Visualizing multiple sources of information about a set of sequences together provides an overview of the components found in the sample. The schematic represents a dataset with sequences from a target species and one cobiont, delineated by dashed lines. Each point represents one sequence, and coordinates reflect two-dimensional representations of tetranucleotide composition learned by a VAE. Size indicates sequence length. Sequences with similar composition cluster together. Colors represent sequence annotations. a) shows available taxonomic labels. The target (left) is displayed in blue, the cobiont (right) in red, and unassigned sequences in gray. b) shows values for sequence statistics (for example, coding density), with lighter colors corresponding to larger values. Where labels are missing in (a), information from (b) can help differentiate distinct components.

In this work, I examine how visualizing annotated low-dimensional representations of sequence k-mer composition can help detect cobionts and contaminants in samples. To address the challenges of working with large read sets, I implemented a variational autoencoder (VAE) (Kingma and Welling 2014) that projects tetranucleotide counts into two dimensions. VAEs have proven useful for a variety of biological applications, from examining population structure to predicting variant effects and protein function (Battey *et al.* 2021; Frazer *et al.* 2021; Boddé *et al.* 2022; David and Halanych 2023) Annotating the two-dimensional embeddings learned by the VAE with additional sequence characteristics, such as estimated coding density, further highlights differences in composition between sequences from different sources (Fig. 1). I also developed a k-mer based method to approximate coverage, modifying an established procedure for read set profiling (Myers 2021). In addition, an interactive dashboard is provided to explore which organisms might be represented in a sample. Rather than attempting to explicitly classify or bin sequences, these tools are intended as part of a multilayered cobiont identification strategy.

Using data from 204 lepidopterans sequenced by the DToL project (Darwin Tree of Life Project Consortium 2022), I illustrate the impact of taking an integrated approach to cobiont detection and demonstrate that the results are consistent with the output of a reference-based decontamination pipeline. Examples from fish, green algae, and plants show that the composition-based approach can be applied to a broad range of taxa. The VAE allows the workflow to scale to large long-read datasets. In addition, it is able to retrieve cobionts where reference-based approaches commonly fail—particularly where no closely related references are available, as in the case of microsporidians or myxozoans. Finally, I illustrate how target yield can be assessed given a heavily contaminated unassembled read set.

## Materials and methods

### Data

The analyses presented here are based on Pacific Biosciences single-molecule HiFi long reads generated by DToL from single specimens. The 204 lepidopteran species considered, including *Phalera bucephala* and *Blastobasis lacticolella*, are described in Vancaester and Blaxter (2023). In addition, reads from *Brachiomonas submarina*, *Viscum album*, and *Thunnus albacares* (from the Vertebrate Genomes Project) were examined. Primary contigs for *P. bucephala*, were assembled with hifiasm version 0.12 using default settings (Cheng *et al.* 2021). This assembly is intended for illustration, and does not reflect the released *P. bucephala* assembly.

### Sequence composition

#### K-mer counts

The primary measure of sequence composition considered in this work are canonical tetranucleotide counts (each k-mer and its reverse complement are assigned the same key). In principle, other k-mer sizes *k* could be used, but k=4 provided a reasonable balance between computational cost and the ability to disentangle sequences for a range of samples. Considering the canonical counts reduces the number of features per sequence from 256 to 136 and ensures that reads and their reverse complements cluster together (though note that the full set of 256 captures information about strand asymmetry). To tally counts efficiently, I implemented a Rust program built on the Needletail library. Memory requirements depend on *k* and sequence length, not the number of sequences, making the implementation suitable for datasets with millions of sequencing reads. The k-mer counter is available at https://github.com/CobiontID/kmer-counter.

#### VAEs for read decomposition

In order to visualize high-dimensional tetranucleotide count vectors, a VAE is used to reduce the data into two-dimensional space (Kingma and Welling 2014, 2019). VAEs consist of a pair of deep neural networks, with an encoder that projects the observed input features (*x*) into a lower-dimensional latent space (*z*), and a decoder that attempts to reconstruct the original input features from samples from the latent space.

Intuitively, sequences with similar tetranucleotide composition will be closer together in latent space than dissimilar sequences. In addition, the decoder’s ability to reconstruct the inputs relies, in part, on the latent space capturing the salient features and structure of the input data. The latent variables can therefore provide information about the processes that generated the observed data (Murphy 2023).

VAEs generally provide better class separation than principal component analysis (PCA), given their ability to learn nonlinear projections (Graves *et al.* 2018; Battey *et al.* 2021). This observation holds for read tetranucleotide composition (Supplementary Fig. 8). They are also more computationally efficient than methods such as t-SNE or UMAP (Van der Maaten and Hinton 2008; McInnes *et al.* 2018), making them suitable for large read datasets.

The encoder network of the VAE $q_{\phi }(z|x)$ does not produce deterministic encodings. Instead, it returns vectors parameterizing the mean (*μ*) and variance of a Gaussian distribution from which random samples are drawn. This makes the model robust to noise in the input data. The decoder $p_{\theta }(x|z)$ then maps the samples back to the original high-dimensional space (see Kingma and Welling 2019, Fig. 2.1). The variational parameters *ϕ* and the decoder parameters *θ* are learned by the respective neural networks.

The network is trained by optimizing the lower bound on the log likelihood of the data (ELBO) (Kingma and Welling 2014), which is given by:


(1) $$L_{\beta }(\theta ,\phi |x)=\underset{\mathrm{reconstruction}\;\mathrm{error}}{\underbrace{E_{q_{\phi }(z|x)}[\log\;p_{\theta }(x|z)]}}-\beta \underset{\mathrm{regularization}\;\mathrm{term}}{\underbrace{D_{KL}(q_{\phi }(z|x)\|p_{\theta }(z))}}$$

The first term of the model’s loss function serves to minimize the reconstruction error, so that the decoder $p_{\theta }(x|z)$ learns to produce outputs that closely resemble the original input *x*. In addition, a regularization term encourages the encoder distribution $q_{\phi }(z|x)$ to be close to the unit Gaussian prior $p_{\theta }(z)$ by minimizing the Kullback–Leibler divergence. The latter ensures a compact latent space, where similar inputs are placed close together.

The weighting factor *β* adjusts the penalty on the regularization term. For the tetranucleotide count data, setting β<1 is necessary to ensure that the model stores useful information about the inputs in the latent space (Wang *et al.* 2021), thus allowing sequences to be visually separated (see Supplementary Fig. 7). Hence, the model is a *β*-VAE (Higgins *et al.* 2017). Results obtained with other model architectures, such as Associative Compression Networks (Graves *et al.* 2018), or VQ-VAE (van den Oord *et al.* 2017), were qualitatively similar. Further details on the implementation of the model and hyperparameter choices are described in the Supplementary Material. Code is available from https://github.com/CobiontID/read_VAE.

#### Estimated coding density

Given expected differences in coding density between taxonomic groups (low and heterogeneous in most animals, high and relatively homogeneous in bacteria), sequences were additionally annotated with estimated coding densities using hexamer (Durbin and Thierry-Mieg 1994), which identifies putative blocks of coding sequence by comparing the composition-controlled 6-mer statistics of windows in a sequence to a reference trained on protein coding sequences. We modified the program to process multirecord fasta files and return the sum of the length of the predicted coding sequence for each record (enabled by setting the −S flag) (Durbin 2021). The estimated coding density is then given by the ratio of the sum of the length of the predicted coding sequences and the total sequence length (values may exceed 1, as both strands are considered). All analyses presented here used the default threshold of 20 and a reference table trained on *Caenorhabditis elegans*.

#### Estimated read coverage

Median k-mer counts across a read set provide an approximation of coverage, provided *k* is sufficiently large that a given substring has a low probability of appearing in a genome multiple times, but small enough to accommodate the sequencing error rate. The median number of times each k-mer in the query sequence appears across the whole set can be extracted from k-mer profiles generated by FastK (Myers 2021). Here, k=31, given that compatible tables are routinely produced as part of the DToL quality control pipeline. However, k=60 performed similarly. Note that this approach is not suitable for uncorrected reads with higher error rates, such as uncorrected PacBio CLR or ONT reads. The software used to calculate median counts is available at https://github.com/CobiontID/fastk-medians.

#### Unique k-mers

To help distinguish between reads that belong to DNA molecules that are present in many copies (that is, at high coverage), and reads that contain repetitive sequences, k-mer diversity can be considered in addition to median k-mer counts. To calculate this measure, the number of distinct noncanonicalized k-mers in each sequence was counted and divided by the total number of k-mers in the sequence. To ensure that the maximum possible number of distinct k-mers cannot be smaller than the sequence length, *k* is set so that $4^{k}$ is larger than the expected sequence length of a HiFi read (k=8). The counting software is available at https://github.com/CobiontID/unique-kmer-counts.

### Visualizing and exploring reads

Given the large number of reads in each dataset, Datashader (Bednar *et al.* 2021) was used to render the reduced tetranucleotide data efficiently. Unless otherwise noted, the figures included in this work show the encoder outputs *μ*, which provide sharper cluster boundaries than the latent samples. The *x*-axis represents the first latent dimension, and the *y*-axis represents the second latent dimension. To visualize additional sequence statistics such as coding density, continuous values are binned into quantiles and used to color-code each point. Categorical values, such as taxonomic classifications, can be displayed in a similar manner.

**Fig. 2.**
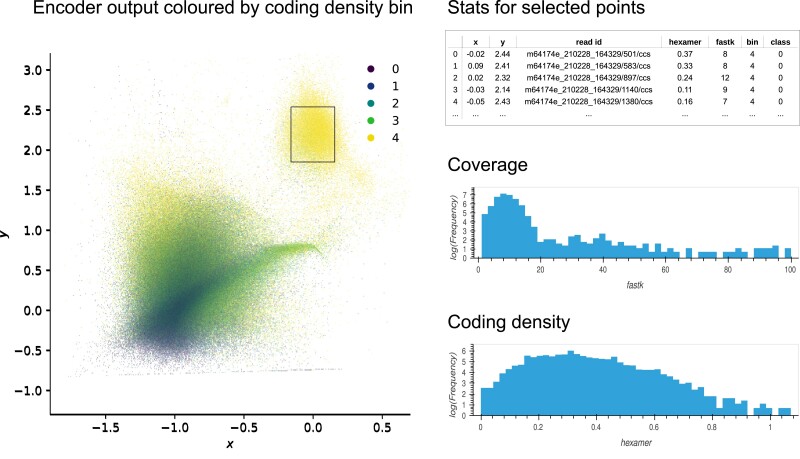
A schematic overview of the interactive dashboard. The left panel displays a color-coded scatterplot of the 2D coordinates returned by the encoder, which can be filtered by coverage and class (data from *Apamea monoglypha*). The interface permits labeling points by coding density bin, as shown here, or class labels (for example taxonomy). The panels on the right show a summary of the data in the selection defined by the rectangular box. Controls for filtering the data and launching blast queries are not shown here for simplicity.

In addition, I provide a Panel (Rudiger *et al.* 2023) dashboard to interactively filter and explore the data (Fig. 2). The interface allows the user to zoom in on and select regions of interest, display and download statistics about the selection, inspect sequences, and launch BLAST queries to allow rapid “spot checking” of read clusters (a local server with the reference database loaded into memory ensures fast turnaround times). Rather than explicitly embedding estimated coverage along with the composition feature vector, the interface provides the option of selectively displaying reads within a specified k-mer coverage range. Reads may also be filtered based on class labels. An interactive demo, complete with an example dataset, is available at https://cobiontid.github.io/reads.html.

### Comparing ascertainment statistics

To examine the circumstances under which sequence composition is helpful in detecting cobionts and contamination, we can consider how often it allowed detection of organisms reported by other tools routinely applied in the DToL assembly and curation process, including NCBI megablast and BTK (see Howe *et al.* 2021, Table 1). In addition, results from MarkerScan were considered (Vancaester and Blaxter 2023, 2024). Briefly, MarkerScan uses a profile HMM to search contigs from a sample of interest for small ribosomal subunit (SSU) sequences, and then uses the taxonomic classifications of the SSUs to construct a streamlined Kraken2 database to classify the reads (Wood *et al.* 2019). Candidate cobiont contigs are identified based on whether they are adequately covered by the classified reads, and additional reads mapping to contigs that meet the threshold are identified. Only cobionts with well-covered contigs are recorded, to distinguish them from horizontal transfers.

For the purposes of this analysis, “detection” was defined in terms of being able to visualize clusters and assign taxon labels by manual spot-checking of the reads, and should not be interpreted as classification performance (inconsistency between samples is also expected). Static plots annotated with estimated coding density, estimated coverage and the number of unique k-mers per base helped identify regions of interest (see Supplementary Fig. 1). In addition, filtering by coverage allowed low-abundance components to be located in the interactive view. Plots were not annotated with taxonomic labels for this analysis.

To automatically identify sequences in different regions of the latent space, reads near local peaks in the two-dimensional histogram of the encoder outputs *μ* were randomly sampled (n=2 for each peak), and queried against the NCBI nt database with blast (version 5, 26th June 2021). Peaks were identified by applying the topology method in the *findpeaks* package (Taskesen 2020) to an equalized histogram with 200 bins, with a window size of 20.

Where possible, overlaps in the recorded organisms were checked at the family level. However, assignments generated during curation were often above the family level, given that they represent a summary of multiple different screens. Therefore, where the family was not retrievable, the next-highest available level was considered. In order to remove redundant entries, a taxonomic tree was constructed from each set of identifiers, and only nodes with no descendants were retained (hence [*Wolbachia*, *Rickettsiales*] simplifies to [*Wolbachia*]). The NCBI Taxonomy (Schoch *et al.* 2020), as implemented in ete3’s NCBITaxa (Huerta-Cepas *et al.* 2016), was used to map between levels (database downloaded 2022 October 3).

For comparisons with Mash Screen (Ondov *et al.* 2019), a lenient identity threshold of 0.9 was set. Frequent spurious Mash Screen hits to species with extremely low GC content, none of which were confirmed by a second method, were discarded (see Supplementary Material). Since viruses are not amenable to classification using conserved marker genes and are often integrated into the host genome, they are not included in the overall comparison.

## Results

### Sequence composition separates genomes from different sources

**Fig. 3.**
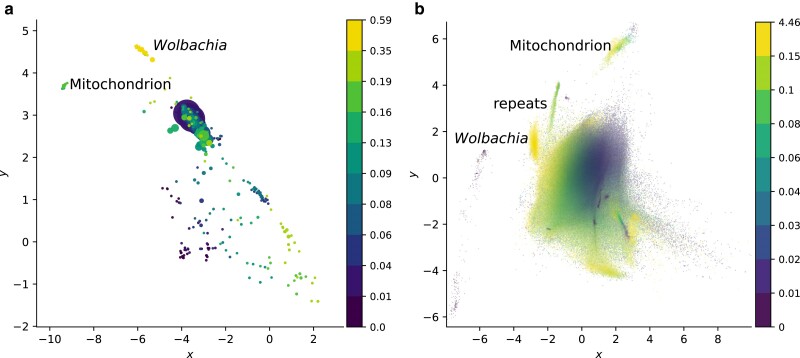
Two-dimensional representations of tetranucleotide count vectors for primary contigs a) and reads b) from the buff tip moth illustrate compositional differences between sequences from different sources. The *x*- and *y*-axes represent the first and second latent dimensions of a VAE. Each point represents one sequence, and is colored according to estimated coding density. Each color represents one decile bin (note that the first two deciles in b) are combined due to the large number of zeros). Markers in a) are scaled according to contig size. Since contigs and reads were embedded separately, the latent spaces do not align, though the relative relationships between components are similar. The larger moth genome is less coding dense and shows more compositional heterogeneity than the *Wolbachia* genome found in the same sample. The small, homogeneous mitochondrial genome shows clear separation from the nuclear genome. *Wolbachia* sequences are located at around (−5.7, 4.5) in (a) and (−2.8, 1.5) in (b). The mitogenome is found at approximately (−9.4, 3.7) in (a) and (2.3, 5.7) in (b).

To explore how projecting tetranucleotide composition into two dimensions with a VAE allows us to separate different sequence components, I first consider the buff tip moth, *P. bucephala* (Boyes *et al.* 2022), which contains three *Wolbachia* endosymbiont genomes in addition to the target’s nuclear and mitochondrial genomes (Vancaester and Blaxter 2023). Primary contigs belonging to the mitochondrial and *Wolbachia* genomes each form tight clusters that are readily distinguished from the lepidopteran contigs (Fig. 3a). In addition to being more homogeneous than the moth sequences, they have notably higher estimated coding density, consistent with a more compact genome architecture.

Can this approach be extended to unassembled reads? As with the contigs, distinct mitochondrial and bacterial read clusters are apparent (Fig. 3b). Read mapping confirms that sequences from the moth nuclear genome, the mitochondrion, and *Wolbachia* are separable in two dimensions (see Supplementary Fig. 1d). The lepidopteran sequences again show more overall heterogeneity, forming multiple clusters.

As expected, *Wolbachia* has overall high coding density, while the moth displays a wider range, reflecting different genomic compartments. In addition, examining the latent representation of the tetranucleotide data highlights a number of groups of repetitive sequences with low k-mer diversity in the moth, including a subset of reads belonging to the heterochromatic female W chromosome (Sahara *et al.* 2012) (see Supplementary Fig. 1). Hence, decomposed tetranucleotide counts can separate both sequences from different taxa, and sequences from the same genome with different characteristics.

Which features are responsible for separating the different components in the sample? The second latent dimension shows a strong correlation with GC content (ρ=−0.98,P<0.0001). However, neither dimension alone separates *Wolbachia* and moth sequences (Fig. 3b), lending support to the observation that GC is useful but, in some cases, insufficient for identifying contamination. Other samples showed a similar pattern, with GC strongly predicting one latent dimension (see Supplementary Table 1, Supplementary Figure 9). The interpretation of the remaining latent dimension is less clear, with no straightforward alignment with any of the other k-mer statistics considered (see Supplementary Figs. 1,2,3,4).

These results confirm that sequences belonging to the organellar genome and endosymbiont stand out based on their composition and inherent genomic features alone. Examining composition ought, therefore, to be useful for separating sequences even where no suitable reference data are available.

### Retrieval of contaminants ascertained by reference-based approaches

To assess how readily cobionts can be detected from two-dimensional representations of read composition, I examined data from 204 butterflies and moths that had previously been screened for contaminants and cobionts (see Vancaester and Blaxter 2023). Lepidoptera are a useful test set, given their known associations with bacterial and fungal endosymbionts, and their relatively small genomes. Routine contamination checks included Mash Screen (Ondov *et al.* 2019) and assessment of scaffolded assemblies with megablast and BTK (Challis *et al.* 2020). In addition, the reads had been classified with MarkerScan (Vancaester and Blaxter 2023). Note that the aim of this analysis was not to assess classification performance, but rather to understand in which settings the approach presented here is likely to be informative.

Overall, composition-based ascertainment of nonlepidopteran sequences via interactive data exploration was high (Table 1). Retrieval of organisms reported by MarkerScan was highest, which is unsurprising as contaminants present at very low coverage are more likely to yield fragmented assemblies with no retrievable SSU region. The sequences of cobionts that were reported by MarkerScan therefore tended to form more conspicuous clusters. Plants and animals, with their heterogeneous genome composition, were difficult to retrieve (but note Fig. 5). Bacterial sequences were also overall more easily located, given that their genomes are relatively homogeneous, leading to more compact two-dimensional representations that are discernible even when coverage is low.

**Table 1.** Ascertainment of cobionts in 204 lepidopterans.

	Fraction of cobionts retrieved relative to			Reads only
	Mash Screen	Curation	MarkerScan	
All	137/162 (85%)	108/135 (80%)	109/121 (90%)	113
Bacteria^a^	136/160 (85%)	97/111 (87%)	97/104 (93%)	94
Fungi	1/1 (100%)	6/11 (55%)^b^	9/10 (90%)	11
Metazoa	–	1/2 (50%)	0/2 (0%)	2
Euglenozoa	–	2/2 (100%)	2/2 (100%)	4
Plant	0/1 (0%)	2/9 (22%)	1/3 (33%)	2

The table gives the fraction of contaminants reported by one of three methods, which could also be detected visually in the latent space of the VAE. Each lepidopteran sample was considered separately, and the counts represent the sums across all species. Contaminants that were apparent in the read embeddings but not reported by another pipeline are shown separately in the last column. ^a^Also see Supplementary Fig. 6. ^b^Four of the records from curation are due to spurious hits, bringing the true fraction to 6/7 (86%).

Given their compactness, we might expect fungi to behave similarly. At first glance, fungal sequences recorded during the curation process appeared to be surprisingly difficult to retrieve. However, this is likely explained by the absence of a sufficiently closely related reference for fungal sequences found in the moth *Apamea monoglypha*. Though the majority of fungal scaffolds in the sample were assigned to the order *Hypocreales*, consistent with results from MarkerScan and the read data, curation records included four additional orders belonging to the class *Sordariomycetes*. Closer inspection revealed these hits (reported by BTK) to be spurious, and there was no evidence that the corresponding scaffolds were compositionally distinct from those labeled as *Hypocreales* (*A. monoglypha* reads are shown in Fig. 2). This alone accounts for 4 of 5 “missing” fungi.

Hence, the apparent inconsistency in performance is not due to limitations of the composition-based approach. In fact, the reference-based approach used in curation failed to detect a number of fungal cobionts due to limited reference data. Due to the lack of significant hits, the problem carried over to the BTK screen. MarkerScan and read visualization were the only approaches consistently able to detect samples infected with microsporidians, which belong to the fungal kingdom, prior to a taxon-specific screen being included in the curation pipeline. As expected, Mash Screen, which relies on exact k-mer matches, performed poorly on nonbacterial cobionts and contaminants (see Table 1). These organisms are less well-represented in reference databases than prokaryotes and often contain a higher fraction of weakly conserved sequence. Of note, Mash Screen rarely detected lepidopteran sequences (hits meeting the threshold were reported in two samples).

Since manually inspecting each sample may not be feasible, we can also consider an automated approach, sampling reads near local peaks in the two-dimensional histogram of the latent distribution (that is, regions with a higher density of points). However, this resulted in a smaller fraction of organisms being identified, as some contaminants do not form sufficiently prominent peaks. Consistent with this, 76% of previously retrieved MarkerScan records were recovered compared to only 57% of Mash Screen records. In other cases, the sampled sequences had no hits in the nucleotide database, reflecting inherent limitations of the reference-based approach. Drawing additional samples could mitigate the issue, at the expense of runtime. Nevertheless, automatically annotating peaks can highlight a subset of cobiont clusters. Additionally, it can flag sequences that stand out compositionally and cannot be assigned to the target genome.

### Retrieval of cobionts is robust to database gaps

The ability of the composition-based approach to detect microsporidians where other methods do not illustrates the advantages of applying unsupervised learning to cobiont screening—especially in cases where the organism infecting the target belongs to a group of organisms with few available genomic resources. However, as noted in Fig. 1, cross-referencing multiple sources of information is a more efficient approach to cobiont identification. To this end, we can annotate tetranucleotide plots with labels generated as part of the MarkerScan pipeline.

**Fig. 4.**
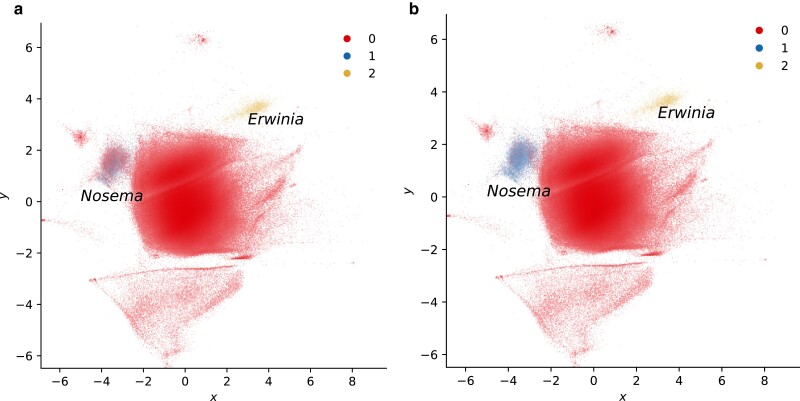
Visualizing decomposed reads illustrates that reference-based methods do not retrieve the full set of nosematid sequences present in the London Dowd moth (*Blastobasis lacticolella*). a) Reads classified as belonging to *Nosema* (blue, (−3.5, 1.5)) and *Erwinia* (gold, (3.4, 3.7)) by Kraken2. Unclassified reads are shown in red. b) Reads classified by MarkerScan by mapping to possible nosematid contigs, resulting in improved retrieval. VAE batch size was set to 32 to improve visual separation.

In the case of *Blastobasis lacticolella*, reference-based approaches failed to reliably identify reads belonging to *Nosema* due to database gaps. While the SSU is readily classified, visualizing the sequences flagged by Kraken2 reveals that around half of the reads in the corresponding cluster remained unassigned (5,523/10,117). Crucially, mapping the unassigned reads in the cluster against *Nosema* contigs confirms that they in fact belong to the microsporidian (Fig. 4). Therefore, combining classification and unsupervised learning can rapidly reveal whether additional undetected cobiont sequences are present.

**Fig. 5.**
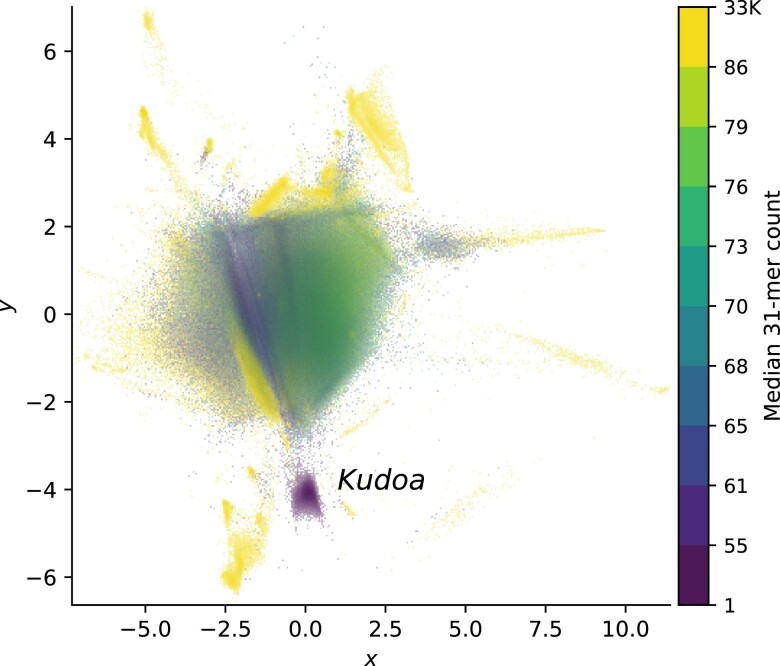
Two-dimensional representations of reads from the yellowfin tuna, *T. albacares*, show evidence of infection with *Kudoa* (located at (0.0, −4.0)). Points are colored by estimated k-mer coverage (k=31), highlighting the difference in coverage between the host and parasite. Repetitive sequences belonging to the host are also apparent.

These observations are not limited to insect–microbe associations. We see a similar pattern in the yellowfin tuna, *Thunnus albacares*, which was infected with a myxosporean parasite of the genus *Kudoa*, a metazoan (manuscript in preparation). Given the large evolutionary distance between the cobiont and the closest relative with an available genome (*K. iwatai*, Chang *et al.* 2015), the fraction of reads classified as *Kudoa* (1,503) was a mere 5% of those that mapped to *Kudoa* scaffolds removed from the host assembly (30,542 reads with a minimum mapping score of 50). In fact, the SSU, present on three scaffolds, was key to identifying the parasite due to low sequence similarity (Vancaester and Blaxter 2024). Though reference-based classification of the *Kudoa* reads performed poorly, they form a conspicuous, tight cluster in the tetranucleotide plot, reflecting a highly reduced genome (Figs. 5, Supplementary Fig. 3).

### Rapid assessment of unassembled read sets

In both examples discussed above, *Nosema* and *Kudoa* stand out, in part, because their estimated coverage differs markedly from that of the host (see Figs. 5, Supplementary Fig. 2). Visualizing k-mer coverage as a third dimension through the use of color can hence highlight nontarget sequences. Histograms summarizing k-mer coverage are a common tool to assess if a sample has been sequenced to sufficient coverage to assemble successfully. They can also reveal whether significant contamination is present (Vurture *et al.* 2017; Ranallo-Benavidez *et al.* 2020). However, in isolation, they provide limited information about where the target falls in the distribution. Given a histogram for a particular sample, how can we confirm if a peak belongs to the organism of interest? Cross-referencing two-dimensional representations of read composition and coverage histograms, labeling corresponding coverage ranges with the same colors, is one possible approach.

**Fig. 6.**
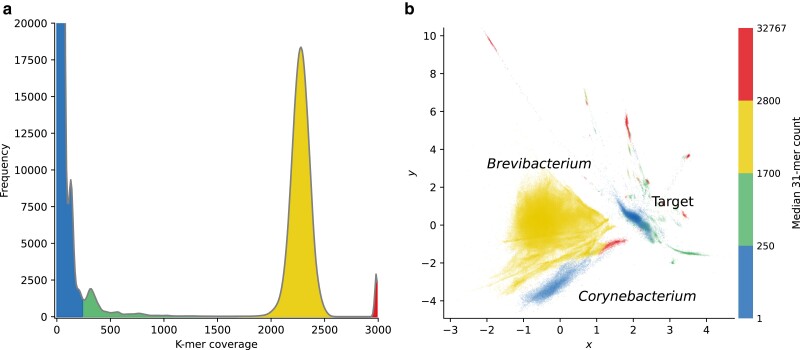
Visualizing the k-mer coverage histogram for the green alga *Brachiomonas submarina* alongside read composition allows clusters corresponding to peaks to be identified. The large peak at 2,300-fold coverage belongs to *Brevibacterium*, a contaminant. a) Count histogram displaying the number of unique k-mers (k=31) that appear at a given coverage in the dataset. Colors indicate four discrete bands chosen to include conspicuous local peaks in the distribution. b) Decomposed read tetranucleotide composition colored according to k-mer coverage bins shown in (a). Note that the k-mer coverage distributions of the target reads (2.0, 0.3) and *Corynebacterium* (−0.3, −3.0) overlap, so they cannot be separated based on coverage alone.

The green alga *Brachiomonas submarina* provides an illustration. A high peak in the k-mer coverage histogram is apparent at around 2,300. However, closer inspection reveals that it is accounted for by sequences from *Brevibacterium linens*, whereas the corresponding peak for the target species is at only around fivefold coverage (Fig. 6). In absence of a preliminary assembly, the identities of the organisms in the clusters can be determined by spot-checking against the NCBI nt database. Sampling reads near local peaks in the two-dimensional distribution confirms that the reads corresponding to the large peak belong to the order *Micrococcales*, with the majority matching the family *Brevibacteriaceae* (see Supplementary Fig. 5). Reads matching the order *Corynebacteriales* are also present in the coverage range overlapping the target’s, but are readily distinguished from the alga based on tetranucleotide composition. Over 85% of reads map to the genome of *Brevibacterium linens strain RS16*, which returned the highest BLAST bitscore for the sampled reads (minimum mapping quality score = 60). Interactively exploring the data leads to similar conclusions.

Although the coverage ranges in this example were assigned manually for illustration, plots showing automatically assigned bins can be similarly informative. Logarithmic coverage bins indicate that the *Brevibacterium* cluster falls into the same range as the high-coverage histogram peak (Supplementary Fig. 4d). In addition, taken together with the observation that Mash Screen reports two bacterial contaminants, the higher coding density estimates for the cluster hint at the sequences being bacterial rather than algal (Supplementary Fig. 4a). Therefore, automatically generated visualizations of read composition can add context to the more commonly used coverage histograms for rapid sample quality control.

### Runtimes and scalability

The *P. bucephala* read tetranucleotide counts took around 18 minutes to compute on a 2.99GHz CPU (AMD EPYC 7,713), with a peak of 5 MB memory usage. Gathering k-mer statistics scales easily to very large read sets. Consider the white mistletoe *Viscum album*, which has 199,755,515 reads: Counting tetranucleotides took 25:42 hours on a single CPU, with a maximum memory usage of 7 MB. Memory usage for estimating the coding density of the mistletoe reads peaked at 8 MB and required 42:01 hours on one CPU (real-world run-times could be reduced by processing batches of reads in parallel).

The VAE required 13:43 minutes and 3,951 MB of memory to run for *P. bucephala* (one CPU, 15 epochs, batch size 256). The main computational bottleneck relates to the size of the k-mer count tables. The canonicalized tetranucleotides for *Viscum album* occupy around 100 GB. This translates to an equivalent minimum RAM requirement if the whole array is loaded into memory while training the VAE. In practice, memory usage can be reduced to around 16 GB by loading the preprocessed data in batches, making it feasible to train the model on consumer-grade hardware, albeit at the expense of speed.

## Discussion

This work illustrates how two-dimensional representations of sequence composition can help to identify and separate sequences from different sources in long-read datasets—even where suitable reference sequences are scarce, and the only annotations available are inherent sequence features, such as coding density. As a result, the approach is suitable for a wide range of organisms, including those that are currently not represented in databases. However, it is most effective when integrated with reference-based screens.

In combination with taxonomic labels from other sources, visualizing read composition can highlight sequences that belong to an organism of interest but were not identified as such (as with the microsporidian in *B. lacticolella*). It can also flag sequences that are compositionally distinct from reads that belong to known components of the sample, or whose taxonomic assignments appear inconsistent with their sequence features. Identifying gaps and errors in output from classifiers can thus improve ascertainment of cobiont sequences and facilitate assembling complete genomes.

How might these ideas be applied in practice? Given a fragmented cobiont assembly, reads that map to taxonomically labeled contigs or scaffolds may be assembled together with unclassified compositionally similar reads. Composition may also be used to extend the set of candidate cobiont contigs, especially where suitable chromatin conformation data and taxonomic assignments are missing. This is likely to be particularly useful for unculturable organisms with compact genomes, such as microsporidians (Cornman *et al.* 2009). Tetranucleotide embeddings are currently being used to help produce more contiguous and complete microsporidian assemblies from DToL samples, as genomes from insect-infecting species are scarce (Khalaf *et al.* 2024). The approach has also facilitated the assembly of two myxozoan genomes from low-level contamination in two infected fish (manuscript in preparation). In other cases, the tools described here have been used to help distinguish between current infection and horizontal transfer of endosymbiont sequences into the host genome (Ebdon *et al.* 2021; Lohse *et al.* 2021, 2022). For example, the butterfly *Lysandra bellargus* contains large microsporidian insertions on the W chromosome. In these instances, the structures of the read sets did not resemble infected samples.

As noted in the introduction, the observation that k-mer counts are useful for separating sequences from different microbes is well established (Dick *et al.* 2009; Alneberg *et al.* 2014). Indeed, some available binning tools also make use of VAEs (Nissen *et al.* 2021; Lamurias *et al.* 2022; Wickramarachchi and Lin 2022). However, the results presented in this work highlight a key consideration for exploring eukaryotic samples. Compositional heterogeneity often results in multiple sequence clusters arising from a single genome, both for reads and assembled sequences (Fig. 3). The results of automated binning procedures, like those commonly applied to sequence embeddings in metagenomics, may therefore not be straightforward to interpret. Consistent with this expectation, over-splitting of bins has been reported for mixtures including eukaryotes (Vancaester and Blaxter 2024). A targeted meta-assembly from a selection of reads of interest may therefore be preferable (Feng *et al.* 2022), with the assembler performing part of the “clustering” task. This has the advantage of reducing the required computational resources compared to a standard meta-assembly, while taking advantage of information not captured by tetranucleotide counts. In line with this idea, considering connections in assembly graphs improves contig binning (Lamurias *et al.* 2022).

Given the presence of repetitive sequences, coverage estimates are also less obviously useful for separating eukaryotic samples. Both map-based contig coverage and read k-mer multiplicity can deviate from the true number of copies of the genome, though the problem is more pronounced for reads. In addition, in many samples discussed here, prokaryotes that were difficult to separate by composition were present at low coverage with overlapping distributions. Given a setting with only two available latent dimensions, the framework presented here therefore does not explicitly embed estimated coverage, unlike some tools designed for microbes (Nissen *et al.* 2021; Lamurias *et al.* 2022; Wickramarachchi and Lin 2022) (see Supplementary Material for further discussion). Instead, coverage is represented using color, and provided as an interactive filtering criterion. Where separating different strains is of interest, the coverage histogram of a region of interest in the latent space can reveal the number of components present (see Fig. 2).

The observations discussed here are based on relatively simple mixtures of sequences: Mostly insect genomes that are overall not heavily contaminated. However, similar principles apply to exploring more complex samples containing many organisms, such as those being cataloged by the Aquatic Symbiosis Genomes Project (McKenna *et al.* 2021). The targets often include eukaryotes from groups that have not been extensively sequenced. Clusters of sequences from associated microbes will nevertheless be relatively easy to identify and extract (see Supplementary Fig. 5). In addition, ensuring sufficient target coverage can be challenging for samples with many components. The expected genome size is often unknown. Annotated tetranucleotide embeddings may hence prove useful in identifying the peaks in the coverage distribution corresponding to the genome of interest.

The VAE model used here produces interpretable outputs across a range of long-read datasets (see https://cobiontid.github.io/vae_tolqc.html for additional examples). However, certain applications and data will benefit from hyperparameter tuning or adjustments to the model architecture. Avoiding posterior collapse, for example, is key to obtaining useful representations (see Supplementary Material) (Wang *et al.* 2021). Identifying the optimal settings for a given dataset is notoriously challenging, particularly in an unsupervised setting (Battey *et al.* 2021). Therefore, the specifics of the implementation should be understood as an example, intended to illustrate how two-dimensional representations can help reveal the structure of a mixture of sequences. Performance on contigs and scaffolds is also less consistent than for reads. Other dimensionality reduction methods may therefore be more suitable for high-throughput applications involving relatively small numbers of assembled sequences.

In practice, explicitly integrating taxonomic information and sequence composition might be more effective than a fully unsupervised approach. Where taxonomic labels are available for a subset of sequences, downstream classification performance could be considered as an additional training objective. In this context, semi-supervised generative models may prove useful (Kingma *et al.* 2014; Makhzani *et al.* 2015). Rather than attempting to bin sequences after the encoder has been trained, they simultaneously learn to embed and classify data, taking advantage of both labeled and unlabeled inputs. This could provide automated assignments while avoiding the drawbacks of pretrained classifiers. With improved reference-based contamination screens capable of handling diverged sequences (Astashyn *et al.* 2024) and more comprehensive reference data, suitable partially labeled sequences will become more readily available in future.

Another consideration is selecting useful input features. The latent variables of the VAE can be interpreted as factors that give rise to the observed data distribution. Codon models, which parameterize protein-level selection and mutation bias (Goldman and Yang 1994; Muse and Gaut 1994; Weber and Whelan 2019), are a perhaps more familiar class of generative model describing the evolutionary forces shaping biological sequences. It is not surprising that the organization of the latent space strongly reflects GC content, consistent with the idea that nucleotide bias is a major driver of genome composition (Singer and Hickey 2000; Warnecke *et al.* 2009). Though the second latent dimension does not have an obvious interpretation, it contributes to separating sequences. Because tetranucleotide counts provide a simplified and incomplete summary of sequence composition, they are unlikely to capture some salient differences between species. Information about the spatial distribution of sequence patterns is wholly absent. It would therefore be interesting to examine if inputs based on DNA language models offer an advantage.

These analyses demonstrate how 2D representations of sequence composition can be combined with reference-based labels to provide an integrated view of the contents of long-read genomic datasets. They also highlight that samples containing eukaryotic genomes require a different approach than prokaryotic metagenomes, given differences in genome structure. The tools presented here are hence intended for exploration, rather than automated binning or classification. VAEs or similar classes of generative model can, however, also provide a framework for identifying sequence features useful for classification. Beyond sample quality control, read sequence embeddings can help retrieve genomes from undersampled taxa—even those not originally targeted.

## Supplementary Material

jkae187_Supplementary_Data

## Data Availability

The code for the tools presented here is available from https://github.com/CobiontID/ under an MIT license. Supplementary material available at G3 online.

## References

[jkae187-B1] Alneberg J , Bjarnason BS, De Bruijn I, Schirmer M, Quick J, Ijaz UZ, Lahti L, Loman NJ, Andersson AF, Quince C. 2014. Binning metagenomic contigs by coverage and composition. Nat Methods. 11(11):1144–1146. doi:10.1038/nmeth.3103 25218180

[jkae187-B2] Astashyn A , Tvedte ES, Sweeney D, Sapojnikov V, Bouk N, Joukov V, Mozes E, Strope PK, Sylla PM, Wagner L, *et al*. 2024. Rapid and sensitive detection of genome contamination at scale with FCS-GX. Genome Biol. 25(1):60. doi:10.1186/s13059-024-03198-7 38409096 PMC10898089

[jkae187-B3] Bagheri H , Severin AJ, Rajan H. 2020. Detecting and correcting misclassified sequences in the large-scale public databases. Bioinformatics. 36(18):4699–4705. doi:10.1093/bioinformatics/btaa586 32579213 PMC7821992

[jkae187-B4] Battey C , Coffing GC, Kern AD. 2021. Visualizing population structure with variational autoencoders. G3. 11(1):1–11. doi:10.1093/g3journal/jkaa036 PMC8022710 33561250

[jkae187-B5] Bednar JA , Crail J, Crist-Harif J, Rudiger P, Brener G, Mease J, Signell J, Stevens JL, Collins B, Bird S, et al holoviz/datashader: Version 0.13.0. doi:10.5281/zenodo.4921237

[jkae187-B6] Blaxter M , Archibald JM, Childers AK, Coddington JA, Crandall KA, Di Palma F, Durbin R, Edwards SV, Graves JA, Hackett KJ, *et al*. 2022. Why sequence all eukaryotes? Proc Natl Acad Sci. 119(4):e2115636118. doi:10.1073/pnas.2115636118 35042801 PMC8795522

[jkae187-B7] Boddé M , Makunin A, Ayala D, Bouafou L, Diabaté A, Ekpo UF, Kientega M, Le Goff G, Makanga BK, Ngangue MF, *et al*. 2022. High-resolution species assignment of Anopheles mosquitoes using *k*-mer distances on targeted sequences. eLife. 11:e78775. doi:10.7554/eLife.78775 36222650 PMC9648975

[jkae187-B8] Boyes D , Holland PW, University of Oxford and Wytham Woods Genome Acquisition Lab, Darwin Tree of Life Barcoding collective, Wellcome Sanger Institute Tree of Life programme, Wellcome Sanger Institute Scientific Operations: DNA Pipelines collective, Tree of Life Core Informatics collective, Darwin Tree of Life Consortium. 2022. The genome sequence of the buff-tip, *Phalera bucephala* (Linnaeus, 1758). Wellcome Open Res. 7:28. doi:10.12688/wellcomeopenres.17539.1.

[jkae187-B9] Breitwieser FP , Pertea M, Zimin AV, Salzberg SL. 2019. Human contamination in bacterial genomes has created thousands of spurious proteins. Genome Res. 29(6):954–960. doi:10.1101/gr.245373.118 31064768 PMC6581058

[jkae187-B10] Chakraborty M , Chang CH, Khost DE, Vedanayagam J, Adrion JR, Liao Y, Montooth KL, Meiklejohn CD, Larracuente AM, Emerson J. 2021. Evolution of genome structure in the Drosophila simulans species complex. Genome Res. 31(3):380–396. doi:10.1101/gr.263442.120 33563718 PMC7919458

[jkae187-B11] Challis R , Richards E, Rajan J, Cochrane G, Blaxter M. 2020. Blobtoolkit–interactive quality assessment of genome assemblies. G3: Gen Genom Genet. 10(4):1361–1374. doi:10.1534/g3.119.400908 PMC7144090 32071071

[jkae187-B12] Chang ES , Neuhof M, Rubinstein ND, Diamant A, Philippe H, Huchon D, Cartwright P. 2015. Genomic insights into the evolutionary origin of Myxozoa within Cnidaria. Proc Natl Acad Sci USA. 112(48):14912–14917. doi:10.1073/pnas.1511468112 26627241 PMC4672818

[jkae187-B13] Cheng H , Concepcion GT, Feng X, Zhang H, Li H. 2021. Haplotype-resolved de novo assembly using phased assembly graphs with hifiasm. Nat Methods. 18(2):170–175. doi:10.1038/s41592-020-01056-5 33526886 PMC7961889

[jkae187-B14] Cornman RS , Chen YP, Schatz MC, Street C, Zhao Y, Desany B, Egholm M, Hutchison S, Pettis JS, Lipkin WI, *et al*. 2009. Genomic analyses of the microsporidian nosema ceranae, an emergent pathogen of honey bees. PLoS Pathog. 5(6):e1000466. doi:10.1371/journal.ppat.1000466 19503607 PMC2685015

[jkae187-B15] Darwin Tree of Life Project Consortium . 2022. Sequence locally, think globally: the Darwin Tree of Life project. Proc Natl Acad Sci USA. 119:e2115642118. doi:10.1073/pnas.2115642118 35042805 PMC8797607

[jkae187-B16] David KT , Halanych KM. 2023. Unsupervised deep learning can identify protein functional groups from unaligned sequences. Genome Biol Evol. 15(5):evad084. doi:10.1093/gbe/evad084 37217837 PMC10231473

[jkae187-B17] Dick GJ , Andersson AF, Baker BJ, Simmons SL, Thomas BC, Yelton AP, Banfield JF. 2009. Community-wide analysis of microbial genome sequence signatures. Genome Biol. 10(8):1–16. doi:10.1186/gb-2009-10-8-r85 PMC2745766 19698104

[jkae187-B18] Durbin R . 2021. hexamer. https://github.com/richarddurbin/hexamer

[jkae187-B19] Durbin R , Thierry-Mieg J. 1994. The ACEDB Genome Database. In: Suhai S, editors. Computational Methods in Genome Research. Boston, MA: Springer.

[jkae187-B20] Ebdon S , Mackintosh A, Hayward A, Wotton K, Darwin Tree of Life Barcoding collective, Wellcome Sanger Institute Tree of Life programme, Wellcome Sanger Institute Scientific Operations: DNA Pipelines collective, Tree of Life Core Informatics collective, Darwin Tree of Life Consortium. 2021. The genome sequence of the clouded yellow, Colias crocea (Geoffroy, 1785). Wellcome Open Res. 6:284. doi:10.12688/wellcomeopenres.17292.1. 36157970 PMC9490288

[jkae187-B21] Feng X , Cheng H, Portik D, Li H. 2022. Metagenome assembly of high-fidelity long reads with hifiasm-meta. Nat Methods. 19(6):671–674. doi:10.1038/s41592-022-01478-3 35534630 PMC9343089

[jkae187-B22] Francois CM , Durand F, Figuet E, Galtier N. 2020. Prevalence and implications of contamination in public genomic resources: a case study of 43 reference arthropod assemblies. G3: Gen Genom Genet. 10(2):721–730. doi:10.1534/g3.119.400758 PMC7003083 31862787

[jkae187-B23] Frazer J , Notin P, Dias M, Gomez A, Min JK, Brock K, Gal Y, Marks DS. 2021. Disease variant prediction with deep generative models of evolutionary data. Nature. 599(7883):91–95. doi:10.1038/s41586-021-04043-8 34707284

[jkae187-B24] Galtier N . 2021. Fine-scale quantification of GC-biased gene conversion intensity in mammals. Peer Community J. 1:article no. e17. doi:10.24072/pcjournal.22.

[jkae187-B25] Galtier N , Piganeau G, Mouchiroud D, Duret L. 2001. GC-content evolution in mammalian genomes: the biased gene conversion hypothesis. Genetics. 159(2):907–911. doi:10.1093/genetics/159.2.907 11693127 PMC1461818

[jkae187-B26] Goldman N , Yang Z. 1994. A codon-based model of nucleotide substitution for protein-coding dna sequences. Mol Biol Evol. 11:725–736. doi:10.1093/oxfordjournals.molbev.a040153 7968486

[jkae187-B27] Graves A , Menick J, van den Oord A. 2018. ‘Associative compression networks for representation learning’, arXiv, arXiv:1804.02476, preprint: not peer reviewed. 10.48550/arXiv.1804.02476

[jkae187-B28] Higgins I , Matthey L, Pal A, Burgess C, Glorot X, Botvinick M, Mohamed S, Lerchner A. 2017. Beta-VAE: learning basic visual concepts with a constrained variational framework. In: ICLR.

[jkae187-B29] Howe K , Chow W, Collins J, Pelan S, Pointon DL, Sims Y, Torrance J, Tracey A, Wood J. 2021. Significantly improving the quality of genome assemblies through curation. Gigascience. 10(1):giaa153. doi:10.1093/gigascience/giaa153 33420778 PMC7794651

[jkae187-B30] Hoyt SJ , Storer JM, Hartley GA, Grady PG, Gershman A, de Lima LG, Limouse C, Halabian R, Wojenski L, Rodriguez M, *et al*. 2022. From telomere to telomere: the transcriptional and epigenetic state of human repeat elements. Science. 376(6588):eabk3112. doi:10.1126/science.abk3112 35357925 PMC9301658

[jkae187-B31] Huerta-Cepas J , Serra F, Bork P. 2016. Ete 3: reconstruction, analysis, and visualization of phylogenomic data. Mol Biol Evol. 33(6):1635–1638. doi:10.1093/molbev/msw046 26921390 PMC4868116

[jkae187-B32] Khalaf A , Francis O, Blaxter ML. 2024. Genome evolution in intracellular parasites: microsporidia and Apicomplexa. J Eukaryot Microbiol. e13033. doi:10.1111/jeu.13033 38785208

[jkae187-B33] Kingma DP , Mohamed S, Jimenez Rezende D, Welling M. 2014. Semi-supervised learning with deep generative models. Adv Neural Inf Process Syst. 27.

[jkae187-B34] Kingma DP , Welling M. 2014. Auto-encoding variational Bayes. ICLR.

[jkae187-B35] Kingma DP , Welling M. 2019. An introduction to variational autoencoders. Foundations and Trends® in Machine Learning. 12(4):307–392. doi:10.1561/2200000056

[jkae187-B36] Koutsovoulos G , Kumar S, Laetsch DR, Stevens L, Daub J, Conlon C, Maroon H, Thomas F, Aboobaker AA, Blaxter M. 2016. No evidence for extensive horizontal gene transfer in the genome of the tardigrade Hypsibius dujardini. Proc Natl Acad Sci USA. 113(18):5053–5058. doi:10.1073/pnas.1600338113 27035985 PMC4983863

[jkae187-B37] Kumar S , Blaxter ML. 2011. Simultaneous genome sequencing of symbionts and their hosts. Symbiosis. 55(3):119–126. doi:10.1007/s13199-012-0154-6 22448083 PMC3294205

[jkae187-B38] Lamurias A , Sereika M, Albertsen M, Hose K, Nielsen TD. 2022. Metagenomic binning with assembly graph embeddings. Bioinformatics. 38(19):4481–4487. doi:10.1093/bioinformatics/btac557 35972375 PMC9525014

[jkae187-B39] Lewin HA , Robinson GE, Kress WJ, Baker WJ, Coddington J, Crandall KA, Durbin R, Edwards SV, Forest F, Gilbert MTP, *et al*. 2018. Earth biogenome project: sequencing life for the future of life. Proc Natl Acad Sci USA. 115(17):4325–4333. doi:10.1073/pnas.1720115115 29686065 PMC5924910

[jkae187-B40] Lohse K , Hayward A, Vila R, Howe C, Wellcome Sanger Institute Tree of Life programme, Wellcome Sanger Institute Scientific Operations: DNA Pipelines collective, Tree of Life Core Informatics collective, Darwin Tree of Life Consortium. 2022. The genome sequence of the Adonis Blue, Lysandra bellargus (Rottemburg, 1775). Wellcome Open Res. 7:255. doi:10.12688/wellcomeopenres.18330.1. 37065725 PMC10090861

[jkae187-B41] Lohse K , Mackintosh A, Darwin Tree of Life Barcoding collective, Wellcome Sanger Institute Tree of Life programme, Wellcome Sanger Institute Scientific Operations: DNA Pipelines collective, Tree of Life Core Informatics collective, Darwin Tree of Life Consortium. 2021. The genome sequence of the large white, Pieris brassicae (Linnaeus, 1758). Wellcome Open Res. 6:262. doi:10.12688/wellcomeopenres.17274.1. 36312456 PMC9608253

[jkae187-B42] Makhzani A , Shlens J, Jaitly N, Goodfellow I, Frey B. 2015. ‘Adversarial autoencoders’, arXiv, arXiv:1511.05644, preprint: not peer reviewed. 10.48550/arXiv.1511.05644

[jkae187-B43] McInnes L , Healy J, Saul N, Großberger L. 2018. Umap: uniform manifold approximation and projection. J Open Source Softw. 3(29):861. doi:10.21105/joss.00861.

[jkae187-B44] McKenna V , Archibald JM, Beinart R, Dawson MN, Hentschel U, Keeling PJ, Lopez JV, Martín-Durán JM, Petersen JM, Sigwart JD. 2021. The aquatic symbiosis genomics project: probing the evolution of symbiosis across the tree of life. Wellcome Open Res. 6:254. doi:10.12688/wellcomeopenres.17222.2. PMC12117321 40438199

[jkae187-B45] Merchant S , Wood DE, Salzberg SL. 2014. Unexpected cross-species contamination in genome sequencing projects. PeerJ. 2:e675. doi:10.7717/peerj.675 25426337 PMC4243333

[jkae187-B46] Murphy KP . 2023. Probabilistic Machine Learning: Advanced Topics. Cambridge, MA: MIT Press.

[jkae187-B47] Muse SV , Gaut BS. 1994. A likelihood approach for comparing synonymous and nonsynonymous nucleotide substitution rates, with application to the chloroplast genome. Mol Biol Evol. 11:715–724. doi:10.1093/oxfordjournals.molbev.a040152 7968485

[jkae187-B48] Myers G . 2021. FastK. https://github.com/thegenemyers/FASTK.

[jkae187-B49] Nissen JN , Johansen J, Allesøe RL, Sønderby CK, Armenteros JJA, Grønbech CH, Jensen LJ, Nielsen HB, Petersen TN, Winther O, *et al*. 2021. Improved metagenome binning and assembly using deep variational autoencoders. Nat Biotechnol. 39(5):555–560. doi:10.1038/s41587-020-00777-4 33398153

[jkae187-B50] Ondov BD , Starrett GJ, Sappington A, Kostic A, Koren S, Buck CB, Phillippy AM. 2019. Mash screen: high-throughput sequence containment estimation for genome discovery. Genome Biol. 20(1):1–13. doi:10.1186/s13059-019-1841-x 31690338 PMC6833257

[jkae187-B51] Orakov A , Fullam A, Coelho LP, Khedkar S, Szklarczyk D, Mende DR, Schmidt TS, Bork P. 2021. GUNC: detection of chimerism and contamination in prokaryotic genomes. Genome Biol. 22(1):1–19. doi:10.1186/s13059-021-02393-0 34120611 PMC8201837

[jkae187-B52] Ponsero AJ , Hurwitz BL. 2019. The promises and pitfalls of machine learning for detecting viruses in aquatic metagenomes. Front Microbiol. 10:806. doi:10.3389/fmicb.2019.00806 31057513 PMC6477088

[jkae187-B53] Portik DM , Brown CT, Pierce-Ward NT. 2022. Evaluation of taxonomic classification and profiling methods for long-read shotgun metagenomic sequencing datasets. BMC Bioinformatics. 23:541. doi:10.1186/s12859-022-05103-0 36513983 PMC9749362

[jkae187-B54] Ranallo-Benavidez TR , Jaron KS, Schatz MC. 2020. Genomescope 2.0 and smudgeplot for reference-free profiling of polyploid genomes. Nat Commun. 11:1432. doi:10.1038/s41467-020-14998-3 32188846 PMC7080791

[jkae187-B55] Ren J , Liu PJ, Fertig E, Snoek J, Poplin R, Depristo M, Dillon J, Lakshminarayanan B. 2019. Likelihood ratios for out-of-distribution detection. Adv Neural Inf Process Syst. 32.

[jkae187-B56] Rudiger P , Madsen MS, Liquet M, Artusi X, Hansen SH, Bednar JA, B Chris, Stevens J-L, Signell J, Mease J, *et al*. 2023. Panel. doi:10.5281/zenodo.7590698

[jkae187-B57] Sahara K , Yoshido A, Traut W. 2012. Sex chromosome evolution in moths and butterflies. Chromosome Res. 20(1):83–94. doi:10.1007/s10577-011-9262-z 22187366

[jkae187-B58] Schoch CL , Ciufo S, Domrachev M, Hotton CL, Kannan S, Khovanskaya R, Leipe D, Mcveigh R, O’Neill K, Robbertse B, *et al*. 2020. NCBI Taxonomy: a comprehensive update on curation, resources and tools. Database. 2020:baaa062. doi:10.1093/database/baaa062 32761142 PMC7408187

[jkae187-B59] Singer GA , Hickey DA. 2000. Nucleotide bias causes a genomewide bias in the amino acid composition of proteins. Mol Biol Evol. 17(11):1581–1588. doi:10.1093/oxfordjournals.molbev.a026257 11070046

[jkae187-B60] Steinegger M , Salzberg SL. 2020. Terminating contamination: large-scale search identifies more than 2,000,000 contaminated entries in GenBank. Genome Biol. 21:1–12. doi:10.1186/s13059-020-02023-1 PMC7218494 32398145

[jkae187-B61] Sueoka N . 1962. On the genetic basis of variation and heterogeneity of DNA base composition. Proc Natl Acad Sci USA. 48:582–592. doi:10.1073/pnas.48.4.582 13918161 PMC220819

[jkae187-B62] Taskesen E . 2020. findpeaks is for the detection of peaks and valleys in a 1D vector and 2D array (image). https://erdogant.github.io/findpeaks

[jkae187-B63] Teeling H , Meyerdierks A, Bauer M, Amann R, Glöckner FO. 2004. Application of tetranucleotide frequencies for the assignment of genomic fragments. Environ Microbiol. 6:938–947. doi:10.1111/j.1462-2920.2004.00624.x. 15305919

[jkae187-B64] Vancaester E , Blaxter M. 2023. Phylogenomic analysis of Wolbachia genomes from the Darwin Tree of Life biodiversity genomics project. PLoS Biol. 21(1):e3001972. doi:10.1371/journal.pbio.3001972 36689552 PMC9894559

[jkae187-B65] Vancaester E , Blaxter M. 2024. Markerscan: separation and assembly of cobionts sequenced alongside target species in biodiversity genomics projects. Wellcome Open Res. 9:33. doi:10.12688/wellcomeopenres.20730.1. 38617467 PMC11016177

[jkae187-B66] van den Oord A , Vinyals O, Kavukcuoglu K. 2017. Neural discrete representation learning. Adv Neural Inf Process Syst. 30.

[jkae187-B67] Van der Maaten L , Hinton G. 2008. Visualizing data using t-SNE. J Mach Learn Res. 9:2579–2605.

[jkae187-B68] Vurture GW , Sedlazeck FJ, Nattestad M, Underwood CJ, Fang H, Gurtowski J, Schatz MC. 2017. Genomescope: fast reference-free genome profiling from short reads. Bioinformatics. 33(14):2202–2204. doi:10.1093/bioinformatics/btx153 28369201 PMC5870704

[jkae187-B69] Wang Y , Blei D, Cunningham JP. 2021. Posterior collapse and latent variable non-identifiability. Adv Neural Inf Process Syst. 34:5443–5455.

[jkae187-B70] Warnecke T , Weber CC, Hurst LD. 2009. Why there is more to protein evolution than protein function: splicing, nucleosomes and dual-coding sequence. Biochem Soc Trans. 37(4):756–761. doi:10.1042/BST0370756 19614589

[jkae187-B71] Weber CC , Boussau B, Romiguier J, Jarvis ED, Ellegren H. 2014. Evidence for GC-biased gene conversion as a driver of between-lineage differences in avian base composition. Genome Biol. 15(1):1–16. doi:10.1186/s13059-014-0549-1 PMC4290106 25496599

[jkae187-B72] Weber CC , Whelan S. 2019. Physicochemical amino acid properties better describe substitution rates in large populations. Mol Biol Evol. 36:679–690. doi:10.1093/molbev/msz003 30668757

[jkae187-B73] Wenger AM , Peluso P, Rowell WJ, Chang PC, Hall RJ, Concepcion GT, Ebler J, Fungtammasan A, Kolesnikov A, Olson ND, *et al*. 2019. Accurate circular consensus long-read sequencing improves variant detection and assembly of a human genome. Nat Biotechnol. 37:1155–1162. doi:10.1038/s41587-019-0217-9 31406327 PMC6776680

[jkae187-B74] Wickramarachchi A , Lin Y. 2022. Binning long reads in metagenomics datasets using composition and coverage information. Algorithms Mol Biol. 17(1):1–15. doi:10.1186/s13015-022-00221-z 35821155 PMC9277797

[jkae187-B75] Wood DE , Lu J, Langmead B. 2019. Improved metagenomic analysis with Kraken 2. Genome Biol. 20(1):1–13. doi:10.1186/s13059-019-1891-0 31779668 PMC6883579

