# Supplementary material for: Disentangling cobionts and contamination in long-read genomic data using sequence composition
Source: G3 (Bethesda). 2024 Aug 16;14(11):jkae187. doi: 10.1093/g3journal/jkae187 (PMC11540323; doi:10.1093/g3journal/jkae187)
Supplement: jkae187_Supplementary_Data [file jkae187_supplementary_data.pdf]

Disentangling Cobionts and Contamination in Long-Read Genomic Data using Sequence Composition

Supplementary Material

Read plot annotations

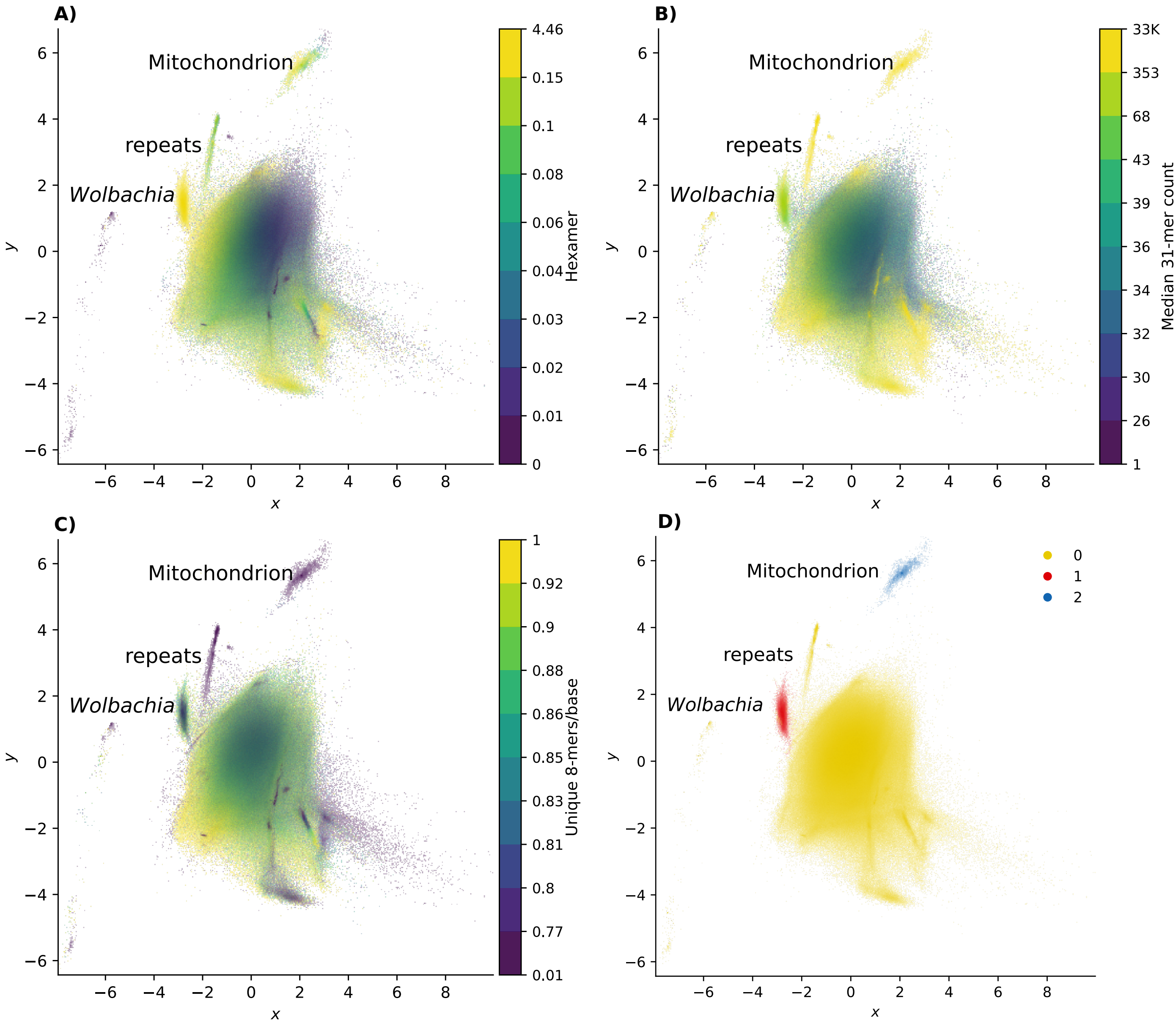

Figure S1 Decomposed tetranucleotide counts for reads from *Phalera bucephala*, annotated according to **A)** estimated coding density; **B)** estimated k-mer coverage ($k = 31$); and **C)** k-mer diversity, measured by the number of distinct k-mers over total number of k-mers ($k = 8$). Low scores indicate sequences that tend to repeat k-mers, accounting for some clusters with high k-mer coverage estimates. The W-linked sequences above the *Wolbachia* cluster, labelled "repeats" in the plots, represent one example of this. **D)** Read mapping with minimap2 (Li 2018) confirms that the VAE model separates sequences from different sources. Reads are colour-coded based on whether they map to the *P. bucephala* nuclear genome (yellow), the *P. bucephala* mitochondrial genome (blue; GenBank: LR990640.1), or one of the three complete *Wolbachia* genomes extracted from the sample (red) (Vancaester and Blaxter 2023) with a score greater than zero. Horizontally transferred sequences, such as NUMTs, group with compositionally similar sequences.

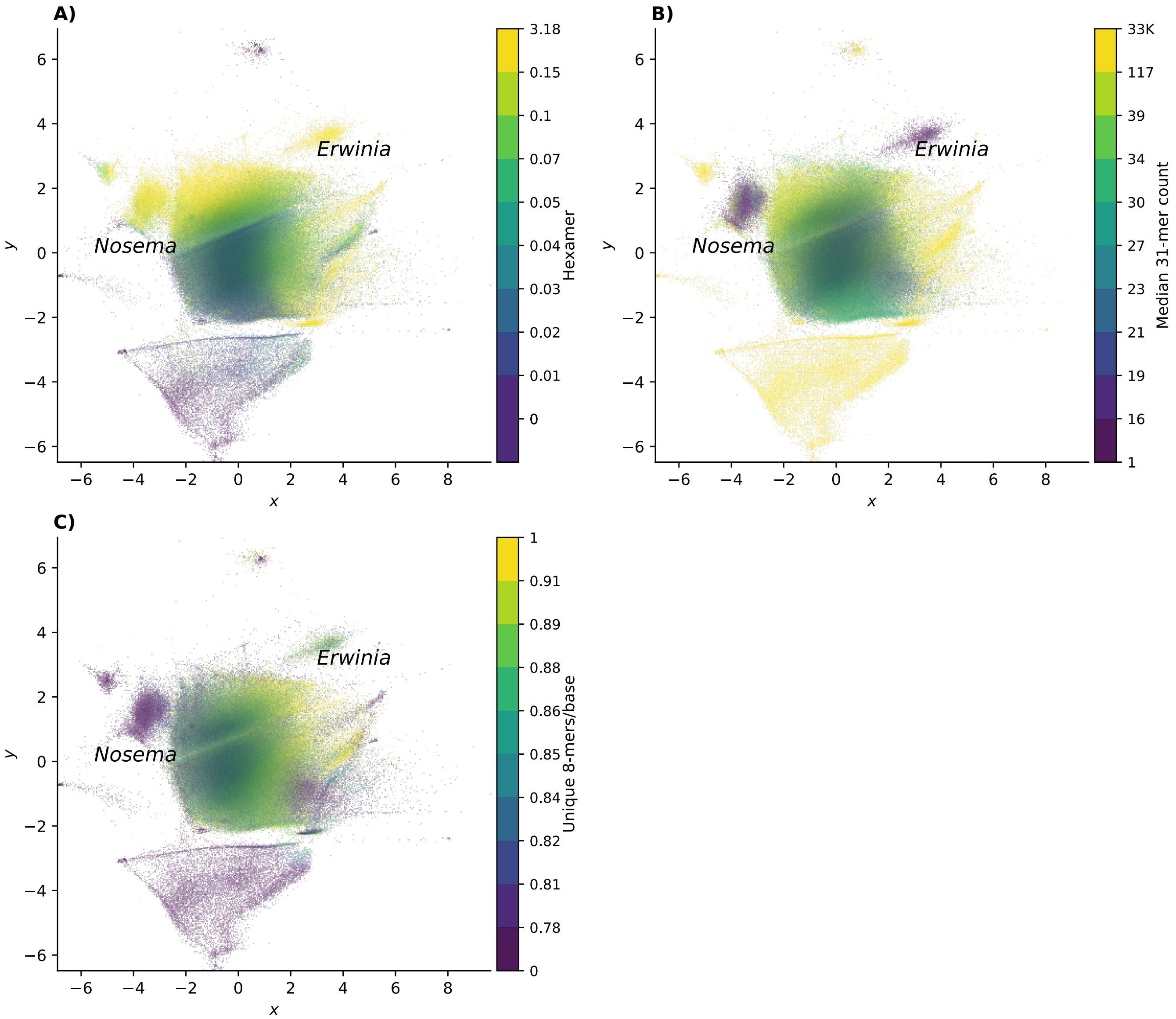

Figure S2 Annotated read plots for *Blastobasis lacticolella*. *Erwinia* and *Nosema* sequences have high coding density and low coverage. *Nosema* sequences have low k-mer diversity, consistent with high genomic AT-content. Note the presence of repeats belonging to the moth with high median k-mer coverage. **A)** Estimated coding density **B)** Estimated k-mer coverage ($k = 31$) **C)** K-mer diversity (number of distinct k-mers/total k-mers for $k = 8$).

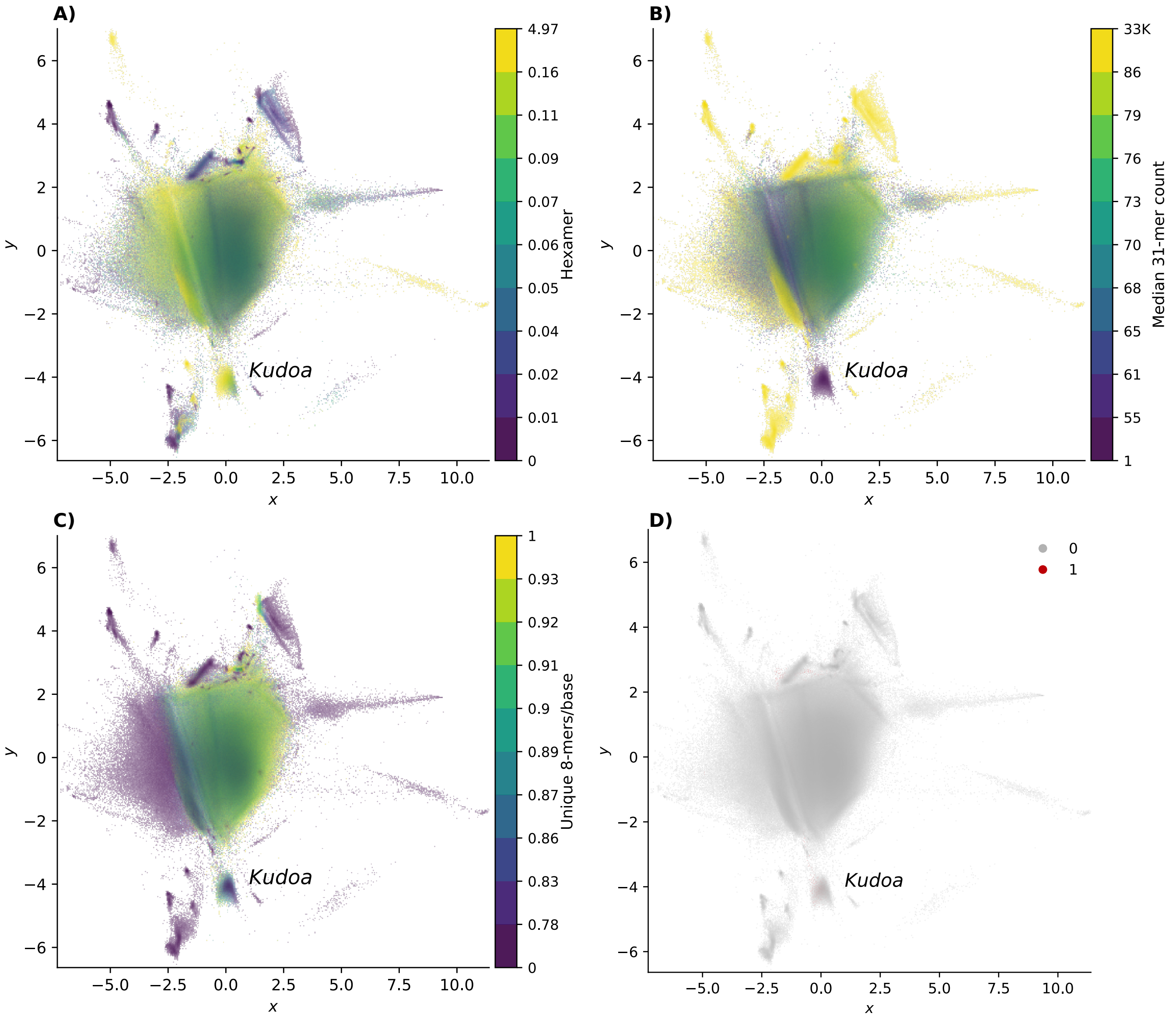

Figure S3 Annotated read plots for the yellowfin tuna, *T. albacares*. **A)** Estimated coding density **B)** Estimated k-mer coverage ($k = 31$) **C)** K-mer diversity (number of distinct k-mers/total k-mers for $k = 8$) **D)** Reads identified as belonging to *Kudoa* by Kraken 2 marked in colour, with the remaining reads marked in grey. The coloured reads are almost imperceptible, highlighting the high false-negative rate.

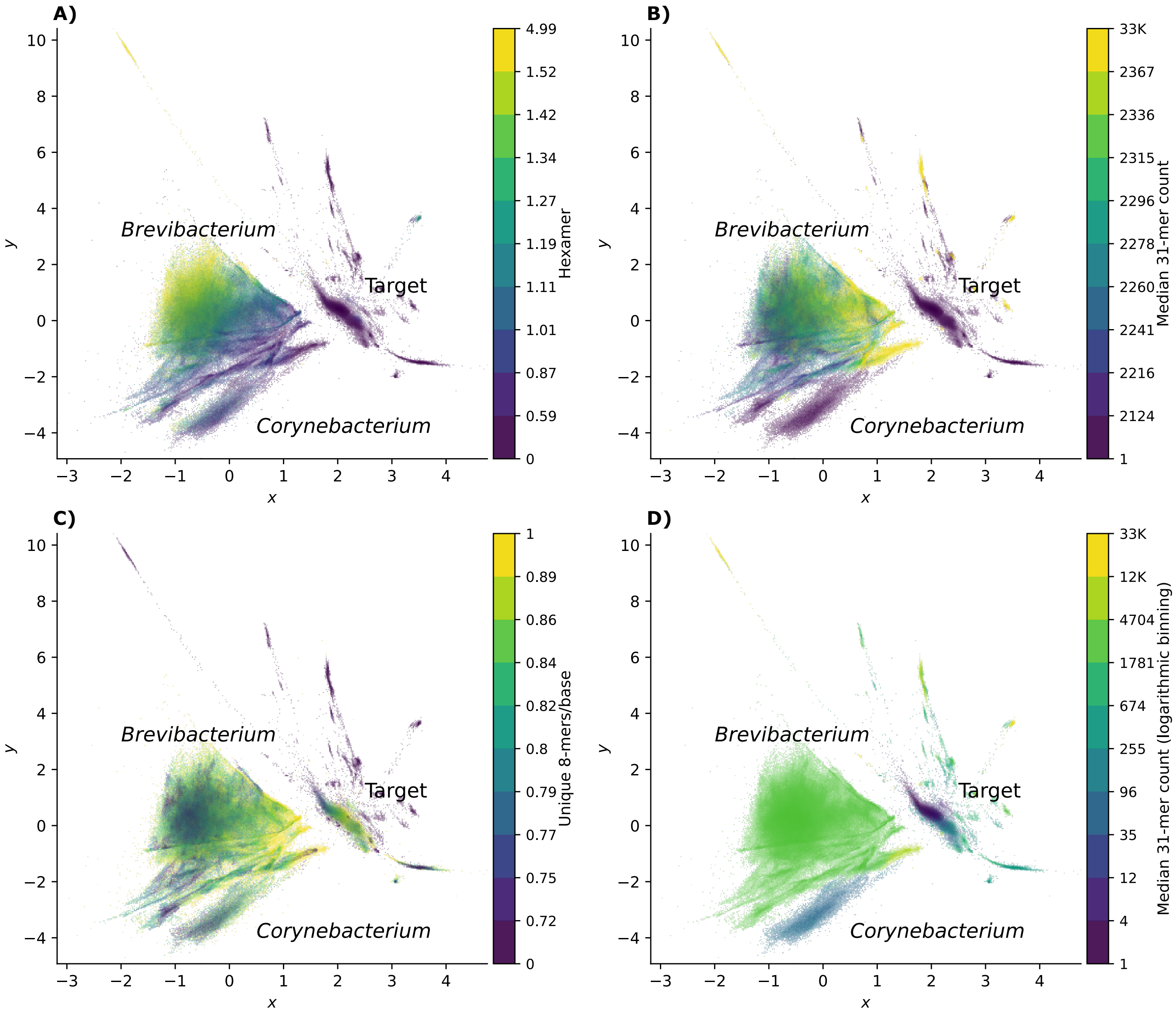

Figure S4 Annotated read plots for *Brachiomonas submarina*. **A)** Estimated coding density **B)** Estimated k-mer coverage ($k = 31$) **C)** K-mer diversity (number of distinct k-mers/total k-mers for $k = 8$). Panels **A**-**C** show decile bins. **D)** Estimated k-mer coverage with logarithmic bins ($k = 31$). Given the k-mer coverage distribution of this sample (Figure 6) discretizing the values using logarithmic equal-width binning provides a more informative overview of the approximate coverage of each component of the mixture.

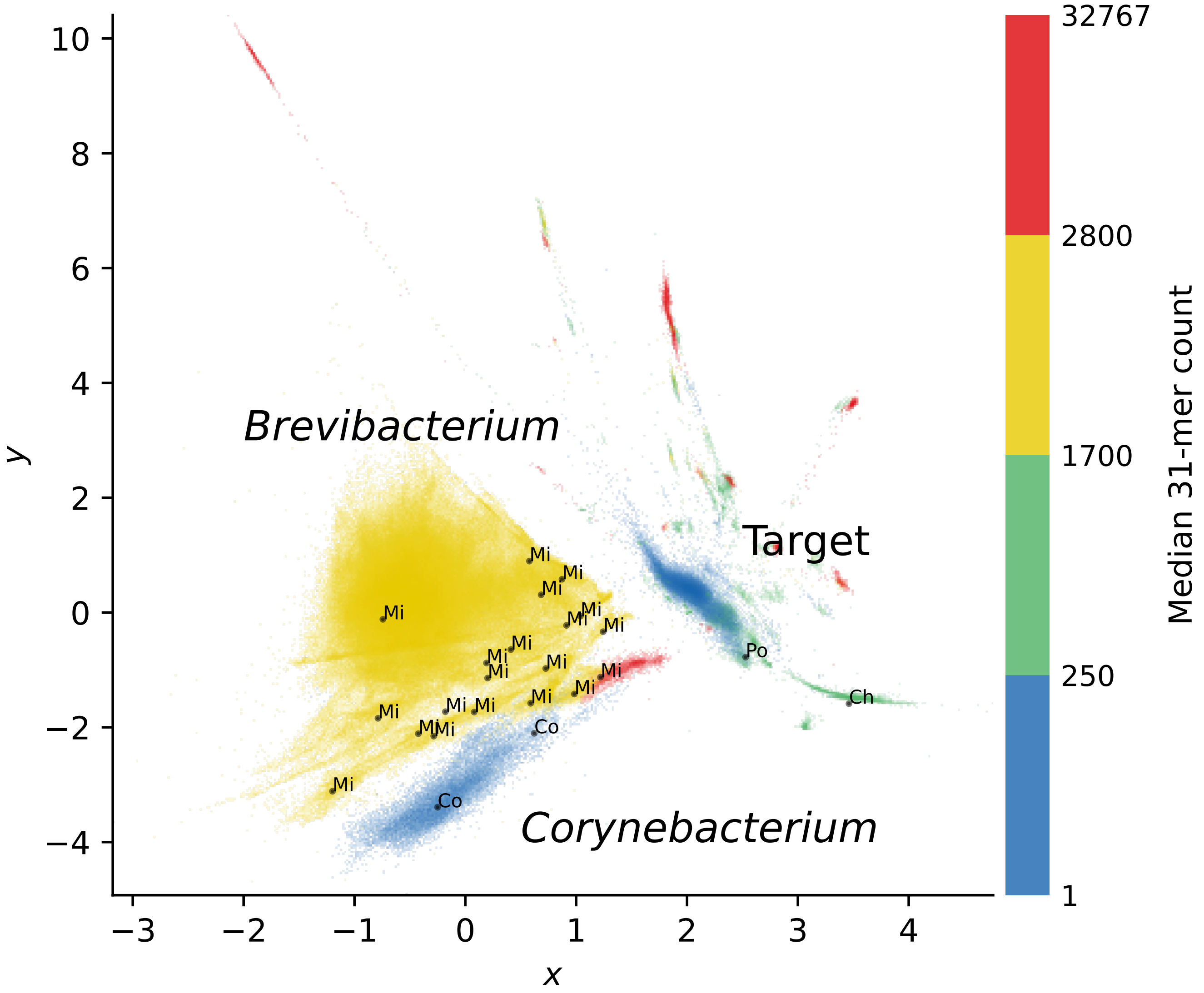

Figure S5 Annotated read plot for *Brachiomonas submarina* showing locations of sampled reads and corresponding nucleotide blast results, labelled by taxonomic order (bins = 100). For each read, the hit with the highest bit score is considered. To simplify the plot, only the first read with a hit is shown for each sampled peak, though results are consistent across all reads. "Mi" denotes hits to *Micrococcales*, "Co" to *Corynebacteriales*, "Ch" to *Chlamydomonadales* (chloroplast), and "Po" to *Poales*. The latter hit is spurious, with a low bit score (178), although it correctly assigns the sequence to *Viridiplantae*, which illustrates the difficulties identifying algal sequences using sparse reference data. The colours represent bands in the k-mer coverage histogram, as in Figure 6.

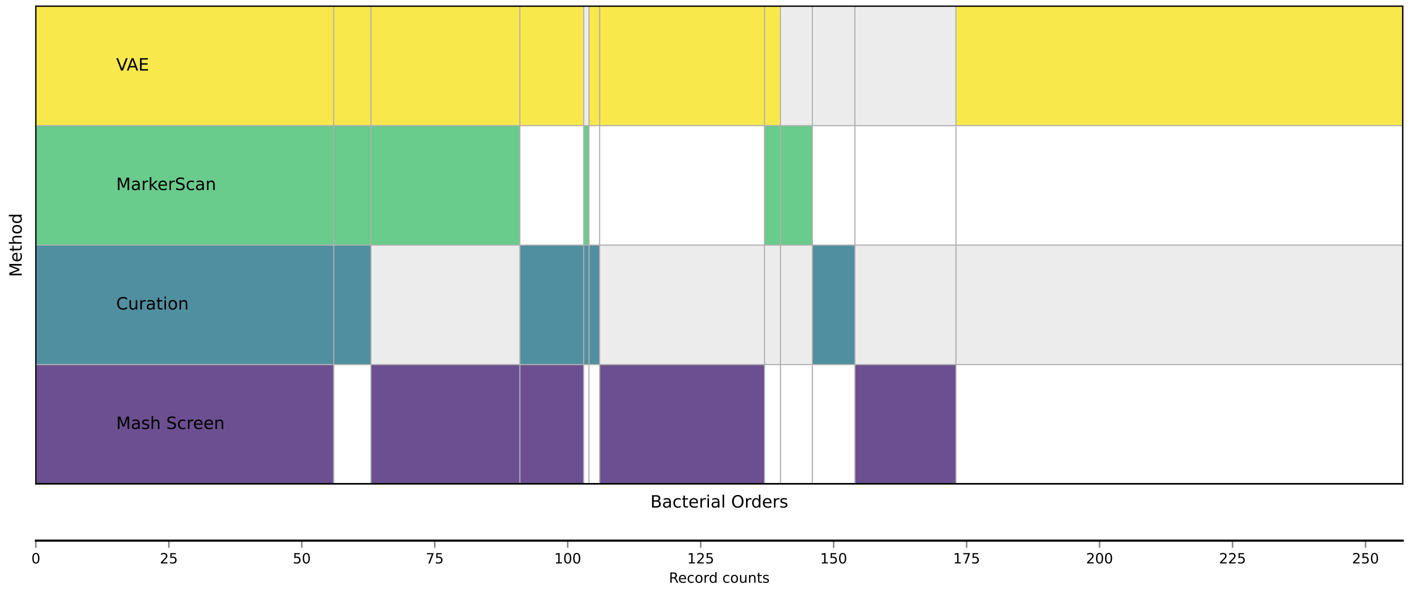

Figure S6 Overlap in bacterial orders between different screening methods. Each row shows one method. Ranges where multiple rows are filled in colour show set overlaps, while unfilled ranges indicate records that are missing relative to other methods. The order of the ranges along the x axis reflects whether a given set is common to all methods (left) or unique to one (right). Note that some organisms recorded during curation are missing from this comparison, as they could not be mapped to an order, and comparisons at a higher taxonomic level would lead to a loss in resolution. These results also differ from those shown in Table 1, where organisms retrieved from the VAE embeddings were mapped to the family level or, if necessary, higher.

VAE implementation and choice of hyper-parameters

The VAE model presented here is implemented using Keras/TensorFlow2 with a custom GradientTape training loop, based on code adapted from https://keras.io/examples/generative/vae/. To account for variation in sequence length, each tetranucleotide feature vector is divided by its sum, and min-max scaling is then applied to each column to give a range between 0 and 1. These scaled vectors are the input X passed to the neural network.

Loss function

As described above, the model objective is given by:

$$\mathcal{L}_{\beta}(\theta, \phi \mid x) = \underbrace{\mathbb{E}_{q_{\phi}(z|x)}\left[\log p_{\theta}(x \mid z)\right]}_{\text{reconstruction error}} - \beta \underbrace{D_{KL}(q_{\phi}(z \mid x) \| p_{\theta}(z))}_{\text{regularisation term}}$$

To compute the reconstruction error term (left), a continuous Bernoulli correction (Loaiza-Ganem and Cunningham 2019) is applied to the binary cross-entropy loss of the reconstruction, to accommodate the bounded distribution resulting from min-max scaling of the length-normalised k-mer count data. The choice of the parameter β that determines the weight given to the regularisation term (right) is described in more detail below ("Mitigating posterior collapse").

Architecture and training

To optimise the model, the encoder and decoder networks, which each contain three hidden layers, are trained end-to-end using the Adam optimiser (Kingma and Ba 2014) for up to 15 epochs with a default mini-batch size of 256, an initial learning rate of 0.001, and a validation split of 0.2. After the first fully connected layer of the encoder network, dropout (p = 0.2) and batch normalisation are applied. This is mirrored in the decoder. The hidden fully connected layers all use a rectified linear unit (ReLu) activation function. The sigmoid activation applied to the decoder's final layer gives a range of outputs between 0 and 1. If the validation loss fails to improve for three epochs, the learning rate is scaled by a factor of 0.2, down to a minimum of 0.00001. In addition, training stops when the validation loss fails to improve for four epochs. The random seed may be fixed, to allow for easier comparison between different hyperparameter settings for a given set of sequences. Plots of the latent embeddings at the start of training and after each epoch can optionally be saved in order to visually track training.

Selecting an optimal set of hyperparameters for a VAE is known to be challenging (Battey *et al.* 2021). Performing an exhaustive grid search for hundreds of read sets is not feasible, particularly given that the model is not supervised - that is, no ground truth is available to evaluate performance against. Because suboptimal solutions that do not capture the underlying structure of the data well can result in a small ELBO (Alemi *et al.* 2018), identifying the settings that result in the smallest loss is insufficient.

Mitigating posterior collapse

A weakness of the VAE is that it is prone to posterior collapse, where one or more latent variables become "inactive", and the encoder $q_{\phi}(z \mid x)$ simply reproduces the prior $p(z)$ (see Murphy (2023)). The model fails to store information that is useful for reconstruction in the latent codes, and therefore z does not capture the underlying structure of the data well. In practice, this results in poor visual separation of dissimilar sequences (Figure S7). One interpretation of this problem is that the KL regularisation term discourages mutual information between the input x and latent representations z (Menon *et al.* 2022; Hoffman and Johnson 2016), and x and z therefore become independent. Posterior collapse may coincide with an especially steep drop in the regularisation loss.

Adjusting the weight β assigned to the term can help avoid parameters that lead to latent collapse (Wang *et al.* 2021). While setting $\beta > 1$ theoretically encourages the model to learn "disentangled" representations, where the latent dimensions each represent a different aspect of the variation found in the input data, it also encourages the model not to use the latent codes. On the other hand, when β « 1, more information tends to be stored in z (Murphy 2023). For the above model and the data considered in this work, setting $\beta = 0.0025$ provides a reasonable balance, with good separation between different sample components, as illustrated in Figure S7. This is coincidentally equivalent to the scaling factor used by VAMB (Nissen *et al.* 2021) for two latent dimensions, while LRBinner (Wickramarachchi and Lin 2022) uses a weight of 0.002.

For some samples, the defaults above nevertheless lead to latent collapse. This is not surprising, as posterior collapse is a function of both the dataset and the model (Wang *et al.* 2021). To mitigate this problem, the implementation described here provides the option to track the mean variance of each latent variable during training (Asperti and Trentin 2020). Values close to one indicate that the latent variable is not informative, and can be set to automatically trigger a reduction in β at the end of the epoch. In many cases, this will "rescue" training and result in a plot that provides at least some insight into the structure of the dataset. Although the callback is not likely to identify the optimal value for β, it provides a mechanism to programmatically identify datasets where the defaults require tuning.

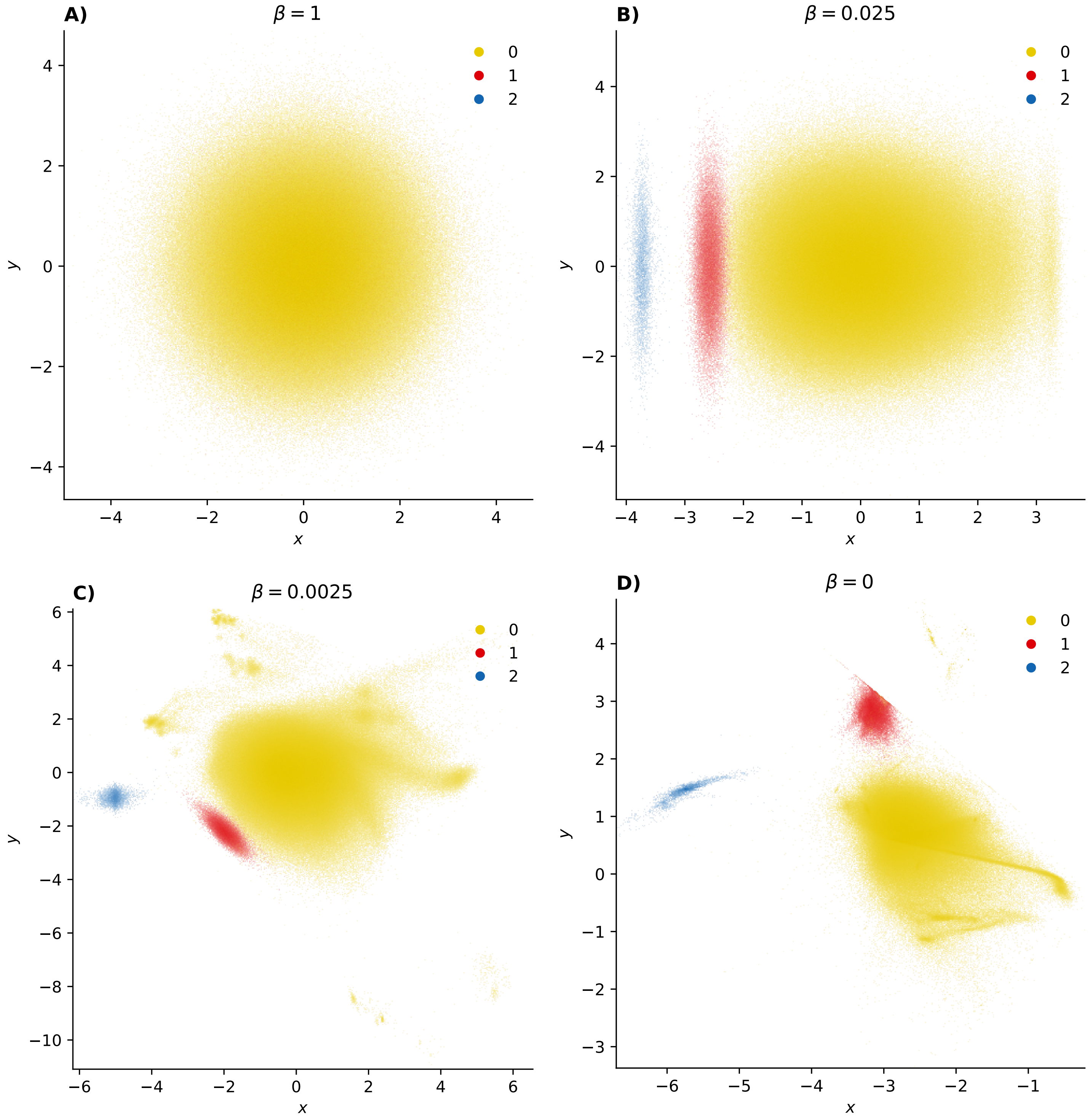

Figure S7 The influence of the weight placed on the regularisation loss on the latent codes z is illustrated with reads from the buff tip moth (*P. bucephala*). Two-dimensional representations of reads are colour-coded based on whether they map to the moth nuclear genome (yellow), the moth mitochondrion (blue), or *Wolbachia* (red), as in Figure S1D. Note that the examples here show z rather than μ, as z better reflects the uncertainty in the latent distribution. **A)** Latent samples generated with $\beta = 1$ (corresponding to the standard VAE) show no separation between groups, as $q_\phi(z|x)$ has collapsed to the prior $p(z)$. **B)** With $\beta = 0.025$, some separation between reads from different sources is apparent along the first latent dimension (x-axis), but fine-scale structure within the moth nuclear genome is not discernible and the second dimension has collapsed. **C)** Samples generated with $\beta = 0.0025$ show separation between sequences from different components of the sample along both latent dimensions, and heterogeneity within the moth nuclear genome is captured. **D)** When $\beta = 0$ (no regularisation), the model imposes no explicit constraints on the latent space. As a result, the data are not centred around zero, and the latent representations are less compact.

Embedding coverage information

Some methods (e.g. VAMB (Nissen *et al.* 2021)) take coverage estimates as input and structure the model so that the decoder reconstructs composition and coverage separately. However, tests on insect data showed no clear practical improvement in sequence separation for two latent dimensions. Here, the base model was extended to embed the natural logarithm of the median k-mer coverage for each read, applying a mean squared error loss to the reconstruction. The coverage vector tended to "take over" one of the two latent dimensions, leaving a single dimension to capture tetranucleotide composition. A very slight advantage could perhaps be noted for small batch sizes (e.g. 16) in some cases. However, reducing the batch size substantially increases the time required to train the model. The "tetranucleotide + coverage" model is, therefore, less suited to high-throughput screening of read datasets that include eukaryotes (see Discussion for limitations of coverage estimates from heterogeneous genomes).

Comparison with PCA

As expected, projecting read tetranucleotide count data into two dimensions using principal component analysis provides some separation between sequences from different sources, but the boundaries tend to be less distinct compared to the latent embeddings from a VAE (see Figure S8). UMAP and t-SNE were too computationally expensive to apply to large read sets, and are therefore not considered here. Therefore, although PCA is fast and can provide a quick snapshot for particularly large datasets, it is insufficient in some cases.

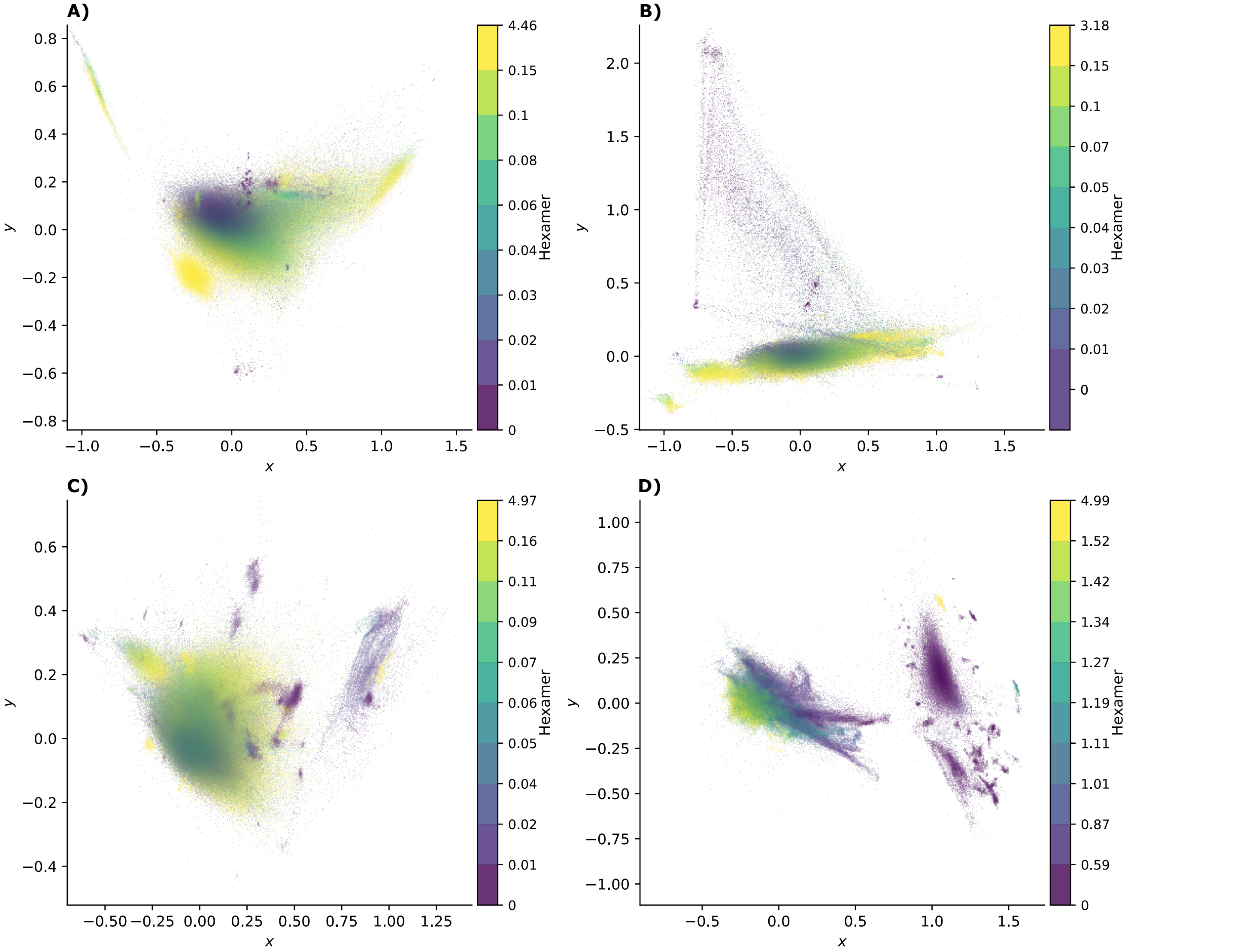

Figure S8 Read tetranucleotide counts from **A)** *P. bucephala*, **B)** *B. lacticolella*, **C)** *T. albacares*, and **D)** *B. submarina* projected into two dimensions by PCA, and annotated with estimated coding density. In addition to producing less distinct read clusters than the equivalent latent embeddings from the equivalent VAE, PCA results in the points being less evenly spread across the plot (see Figures S1 and S2). As a result, the *Erwinia* and *Nosema* clusters in *B. lacticolella* are hardly discernible from static plots. The *T. albacares* sample shows a similar pattern, with poor separation between *Kudoa* and host sequences.

Interpretation of the latent features

Inspecting the latent dimensions revealed that, for all examples illustrated here, GC strongly correlated with at least one latent dimension. Therefore, GC is one feature that contributes to separating sequences from different components of the sample, though it is not sufficient. Where the distribution of GC content overlaps between the target and cobiont reads, as is the case with the buff tip moth and *Wolbachia*, additional information is needed.

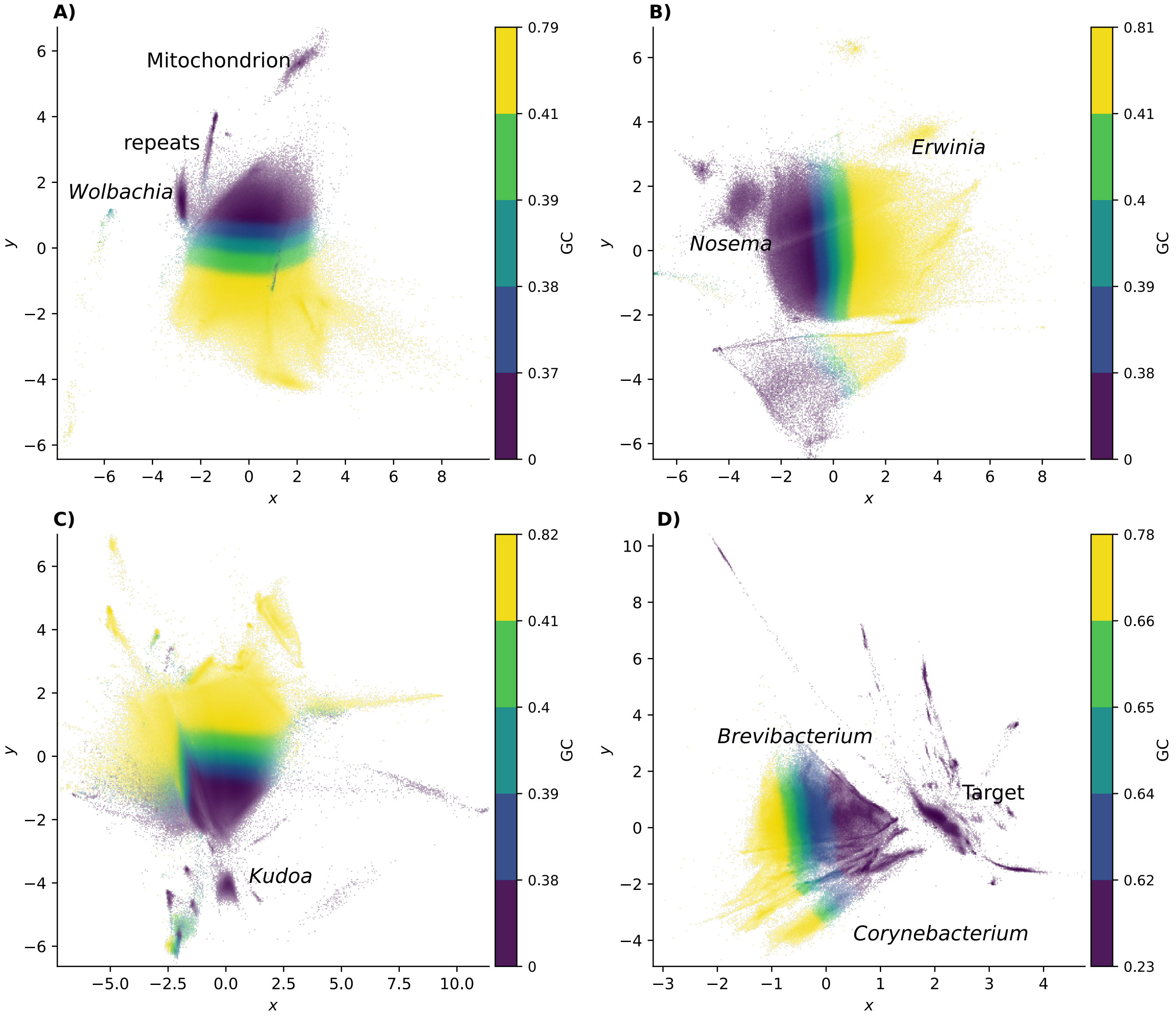

Figure S9 Projected read tetranucleotide counts for *P. bucephala*, *B. lacticolella*, *T. albacares*, and *B. submarina* samples **(A-D)** labelled by GC content quantile. Given the small range of values for intermediate GC contents, only five bins are shown.

Species	Correlation with GC	
	μ_0	μ_1
Phalera bucephala	-0.1	-0.98
Blastobasis lacticolella	0.99	-0.01
Thunnus albacares	0.06	0.94
Brachiomonas submarina	-0.95	-0.18

Table S1 Correlations between GC content and encoder outputs for two latent dimensions, measured by Spearman's rho. $P < 0.0001$ for all correlations. Both dimensions correlating with GC to some extent may be a consequence of $\beta \ll 1$.

Impact of extreme base composition on Mash Screen hits

For many lepidopteran samples, Mash Screen returned spurious hits to *Buchnera aphidicola*, *Candidatus Carsonella ruddii*, *Plasmodium reichenowi*, *Plasmodium gaboni*, *Candidatus Sulcia muelleri*, *Candidatus Phytoplasma oryzae*, *Candidatus Phytoplasma mali*, and *Ichthyophthirius multifiliis*

The case of *Carsonella*, which has a GC content of 14%, illustrates the problem of spurious matches due to biased nucleotide content: Sequences corresponding to sketched k-mer hashes with the default $k = 21$ for the *Carsonella* genome included numerous spurious matches to AT-rich regions of insect contigs. The probability of a random match is given by $\frac{1}{(\Sigma^k/g)+1}$, where g is genome size (see https://mash.readthedocs.io/en/latest/sketches.html). Given that the effective size of the nucleotide alphabet Σ from which the k-mers are drawn is less than 4 when C and G are rare, the default k-mer length of 21 is expected to be too small to provide sufficient specificity. Indeed, re-sketching the *Carsonella* reference with $k = 31$ using Mash Sketch reduces the number of matches in the reads of the *Chloroclystis v-ata* sample from 169 to 1. Meanwhile 126 of 152 matches to *Wolbachia* remain. However, increasing k may lead to an undesirable loss of sensitivity overall. Devising a strategy to mitigate false positives returned by Mash Screen is beyond the scope of this work, and relevant hits were therefore removed.

Literature cited

Alemi A, Poole B, Fischer I, Dillon J, Saurous RA, Murphy K. 2018. Fixing a broken elbo. In: . pp. 159–168. PMLR.

Asperti A, Trentin M. 2020. Balancing reconstruction error and kullback-leibler divergence in variational autoencoders. IEEE Access. 8:199440–199448.

Battey C, Coffing GC, Kern AD. 2021. Visualizing population structure with variational autoencoders. G3. 11:1–11.

Hoffman MD, Johnson MJ. 2016. ELBO surgery: Yet another way to carve up the variational evidence lower bound. In: . volume 1.

Kingma DP, Ba J. 2014. Adam: A method for stochastic optimization. ICLR. .

Li H. 2018. Minimap2: Pairwise alignment for nucleotide sequences. Bioinformatics. 34:3094–3100.

Loaiza-Ganem G, Cunningham JP. 2019. The continuous Bernoulli: Fixing a pervasive error in variational autoencoders. Advances in Neural Information Processing Systems. 32.

Menon S, Blei D, Vondrick C. 2022. Forget-Me-Not! contrastive critics for mitigating posterior collapse. In: . pp. 1360–1370. PMLR.

Murphy KP. 2023. *Probabilistic Machine Learning: Advanced Topics*. MIT Press.

Nissen JN, Johansen J, Allesøe RL, Sønderby CK, Armenteros JJA, Grønbech CH, Jensen LJ, Nielsen HB, Petersen TN, Winther O *et al.* 2021. Improved metagenome binning and assembly using deep variational autoencoders. Nature Biotechnology. 39:555–560.

Vancaester E, Blaxter M. 2023. Phylogenomic analysis of Wolbachia genomes from the Darwin Tree of Life biodiversity genomics project. PLoS Biology. 21:e3001972.

Wang Y, Blei D, Cunningham JP. 2021. Posterior collapse and latent variable non-identifiability. Advances in Neural Information Processing Systems. 34:5443–5455.

Wickramarachchi A, Lin Y. 2022. Binning long reads in metagenomics datasets using composition and coverage information. Algorithms for Molecular Biology. 17:1–15.
